# Supplementary material for: The gut microbiota metabolite trimethylamine N-oxide promotes cardiac hypertrophy by activating the autophagic degradation of SERCA2a
Source: Commun Biol. 2025 Apr 10;8:596. doi: 10.1038/s42003-025-08016-9 (PMC11986001; doi:10.1038/s42003-025-08016-9)
Supplement: Supplementary file 2 — Supplementary information [file 42003_2025_8016_MOESM2_ESM.pdf]

The gut microbiota metabolite trimethylamine *N*-oxide promotes cardiac hypertrophy by activating autophagy SERCA2a degradation

Authors: Dongyu Lei [1,2,3#], Yi Liu[1,2#], Yuan Liu[1,4], Yujie Jiang[2], Yuyan Lei[2,5], Feilong Zhao[2], Wenqun Li[6], Zhonghua Ouyang[7], Lulu Chen[7], Siyuan Tang[8], Dongsheng Ouyang[7], Xiaohui Li[2,7*] and Ying Li[1*]

1 Department of Health Management, The Third Xiangya Hospital, Central South University, Changsha, 410013, China;

2 Department of Pharmacology, Xiangya School of Pharmaceutical Sciences, Central South University, Changsha, 410078, China;

3 Department of Physiology, School of Basic Medicine, Xinjiang Medical University, Urumqi, 830017, China;

4 Department of Anesthesiology, The Second Xiangya Hospital, Central South University, Changsha, 410011, China;

5 Phase Ⅰ Clinical Trial Laboratory, the Second Nanning People's Hospital, Guangxi, China

6 Department of Pharmacy, The Second Xiangya Hospital, Central South University, Changsha, 410011, China;

7 Hunan Key Laboratory for Bioanalysis of Complex Matrix Samples, Changsha Duxact Biotech Co., Ltd., Changsha, 411000, China;

8 Xiangya Nursing School, Central South University, Changsha, 410000, China;

[*]Corresponding author contact details:

Xiaohui Li, PhD, Professor
Department of Pharmacology,
Xiangya School of Pharmaceutical Sciences, Central South University,
Changsha, China
E-mail:xiaohuili@csu.edu.cn

Ying Li, PhD, Professor
Department of Health Management,
The Third Xiangya Hospital of Central South University,
Changsha, China
E-mail: lydia0312@csu.edu.cn.

Supplementary figures

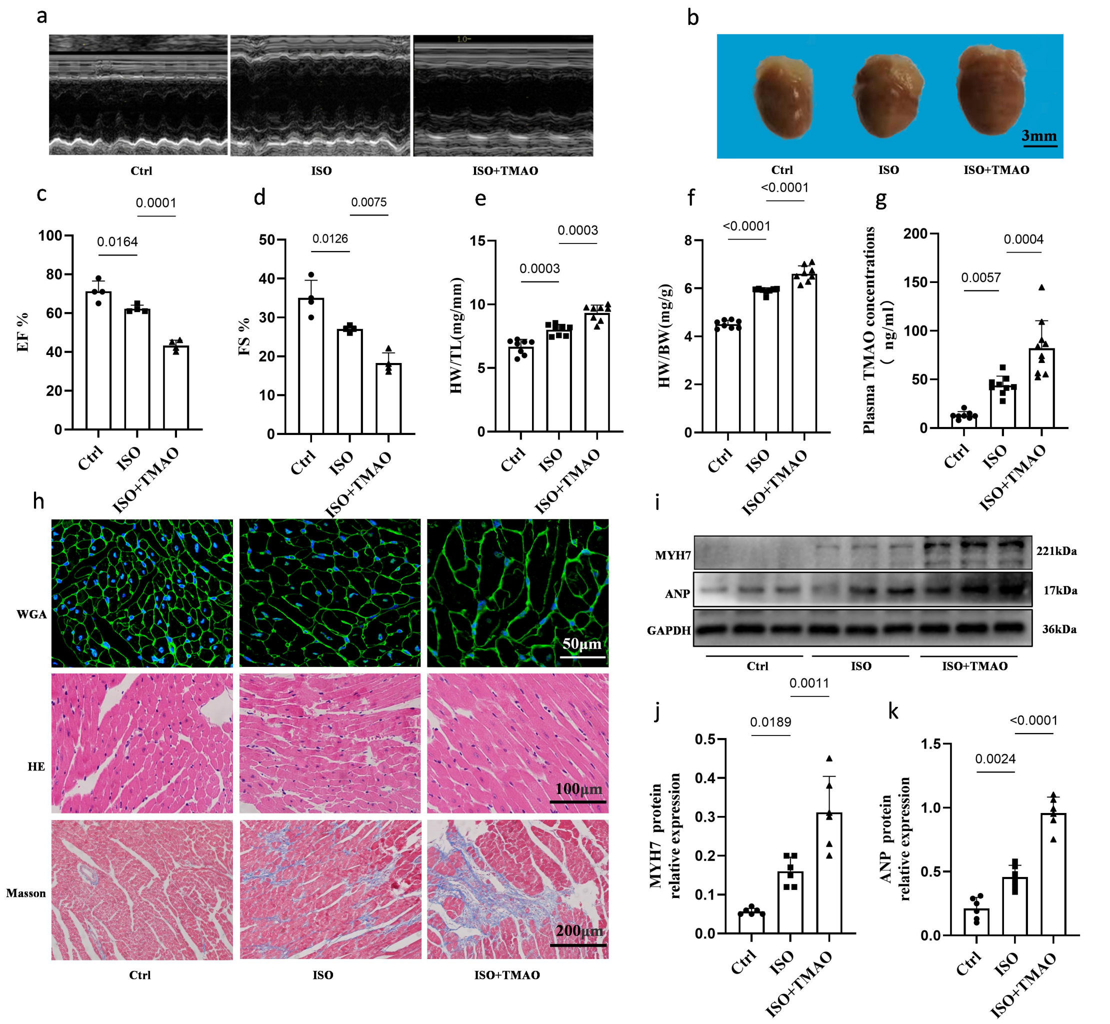

Figure. S1 TMAO exacerbates ISO-induced cardiac hypertrophy.

a Representative images of M-mode echocardiography of the left ventricle. **c-d** Measurement of EF% and FS%, n=4. **b** Representative images of heart size photographed with a stereomicroscope and HW/BW**(e)** and HW/TL**(f)** ratios, n=8. **g** Serum TMAO concentration was determined by UPLC-MS/MS, n=8. **h** Cardiac tissue size and fibrosis were detected by FITC-labeled WGA staining (60×), HE staining (40×), and Masson staining (20×). **i-k** Western blot analysis of ANP and MYH7 in the heart tissues, n=6. Statistical analyse was performed using the One-way ANOVA with Tukey's multiple comparisons test. Error bars represent S.E.M.

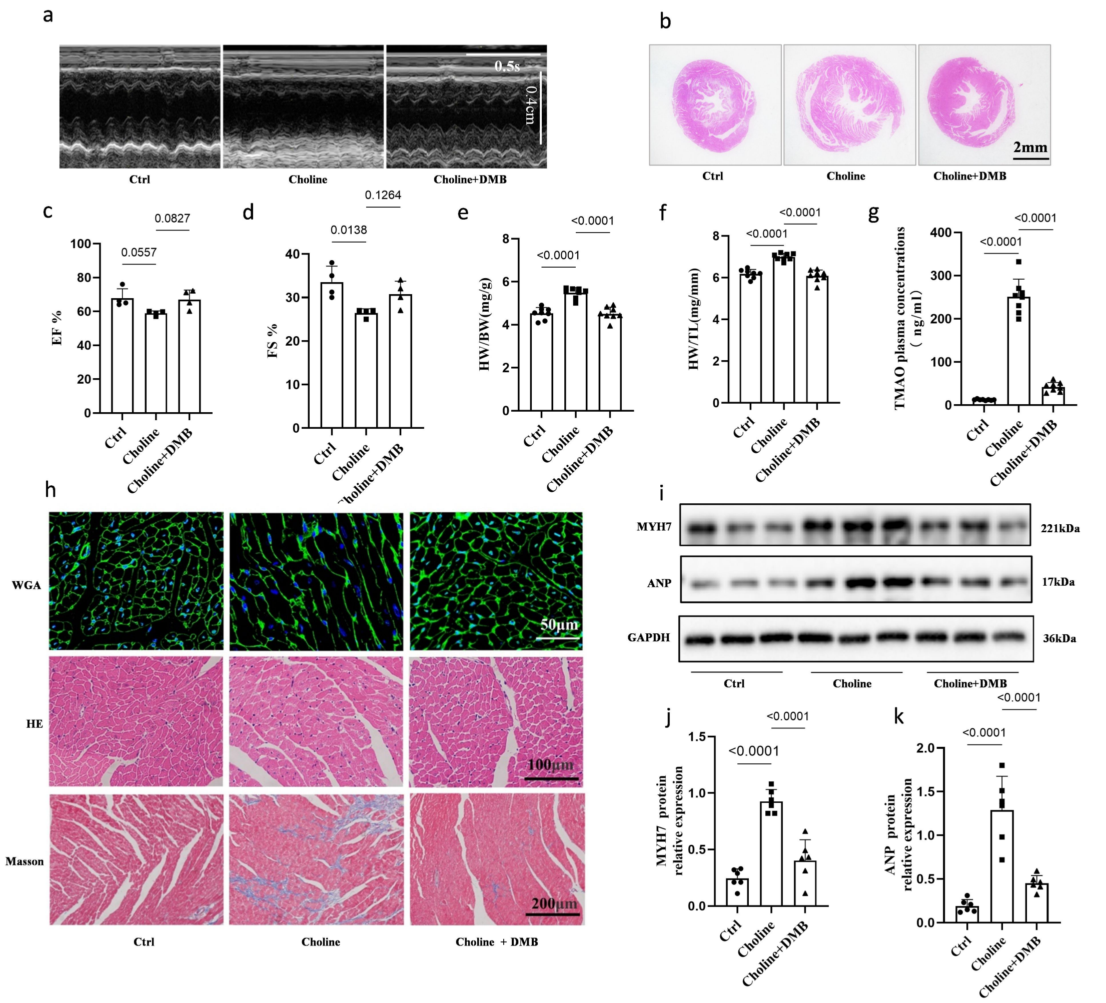

Figure. S2 DMB decreased high choline diet-produced TMAO and reduced cardiac hypertrophy in choline-fed mice

a Representative images of M-mode echocardiography of the left ventricle. **c-d** Measurement of EF% and FS%, n=4. **b** Representative images of heart size photographed with a stereomicroscope and HW/BW**(e)** and HW/TL**(f)** ratios, n=8. **g** Serum TMAO concentration was determined by UPLC-MS/MS, n=8. **h** Cardiac tissue size and fibrosis were detected by FITC-labeled WGA staining (60×), HE staining (40×), and Masson staining (20×). **i-k** Western blot analysis of ANP and MYH7 in the heart tissues, n=6. Statistical analyse was performed using the One-way ANOVA with Tukey's multiple comparisons test. Error bars represent S.E.M.

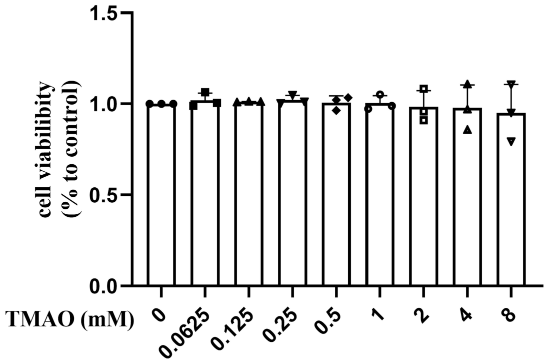

Figure. S3 Cell viability was detected by CCK8 assay in H9c2 cells treated with TMAO (48 hours) in different concentrations, n=3.

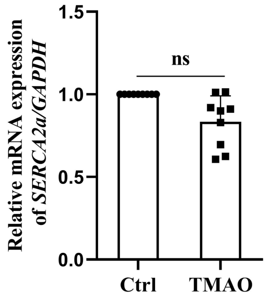

Figure. S4 TMAO does not affect SERCA2a mRNA levels. RT-PCR analysis of SERCA2a in H9c2 cells treated with 1mM TMAO for 48 hours, n=9.

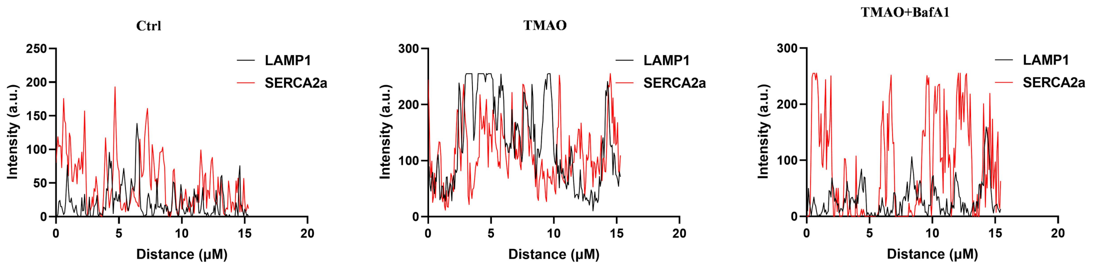

Figure. S5 Relative intensities of immunofluorescence detection of LAMP1 and SERCA2a in H9c2 cells treated with TMAO (1mM) for 24h, followed by treatment with BafA1(10nM) for 24h.

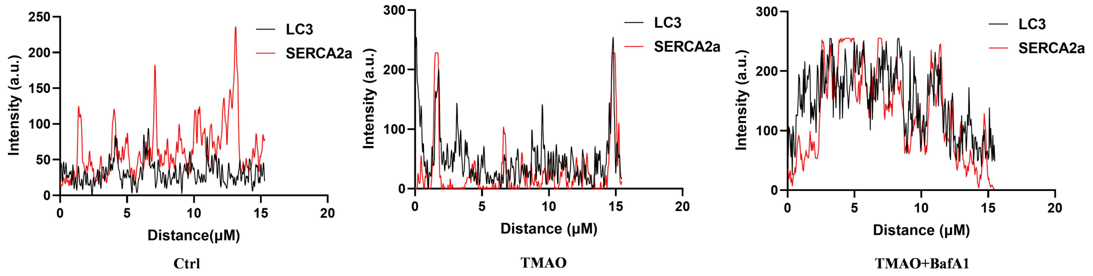

Figure. S6 Relative intensities of immunofluorescence detection of LC3 and SERCA2a in H9c2 cells treated with TMAO (1mM) for 24h, followed by treatment with BafA1(10nM) for 24h.

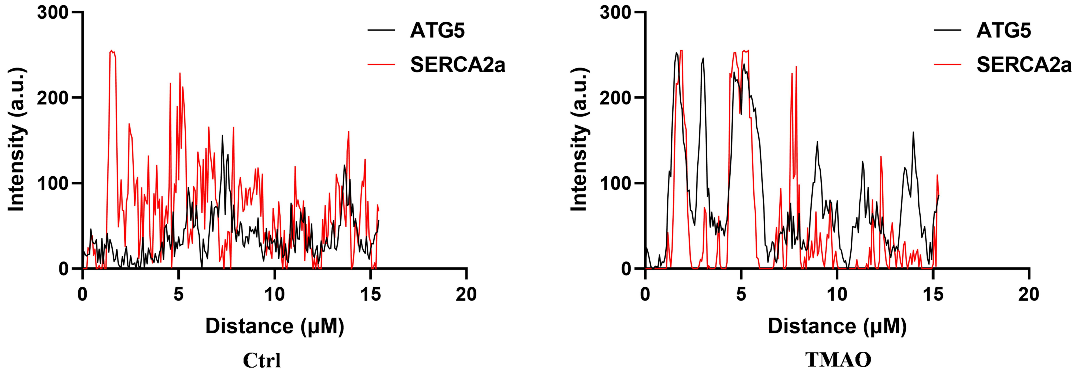

Figure. S7 Relative intensities of immunofluorescence detection of ATG5 and SERCA2a in H9c2 cells treated with TMAO (1mM) for 48h.

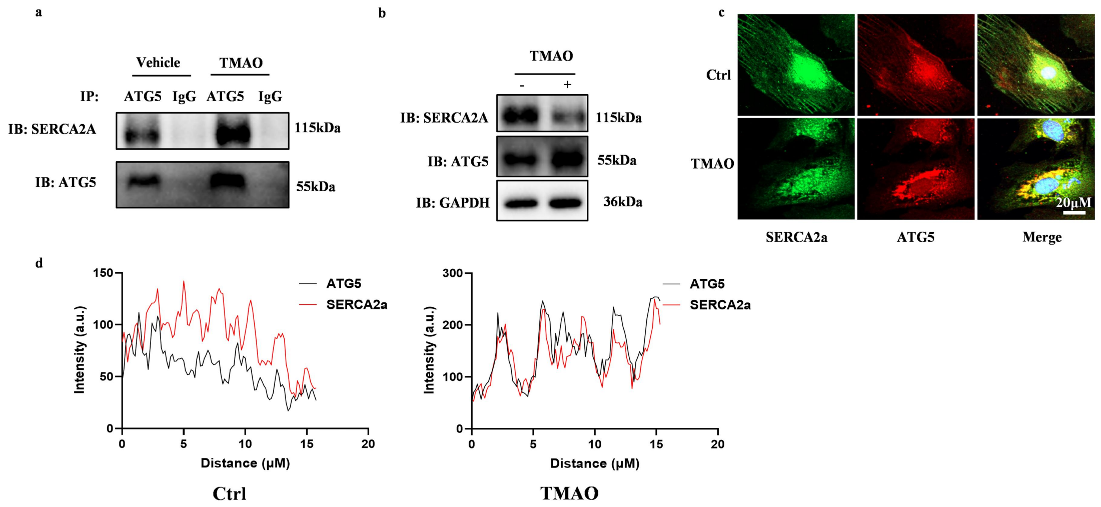

Figure. S8 SERCA2a associates with autophagy protein ATG5 in primary rat cardiomyocytes. **a-b** Primary rat cardiomyocytes were cultured in the presence or absence of TMAO (1mM) for 48h. SERCA2a immunoprecipitation was performed. **c-d** The representative images and relative intensities of immunofluorescence detection of ATG5 (red) and SERCA2a (green) in the presence or absence of TMAO(1mM) for 48h.

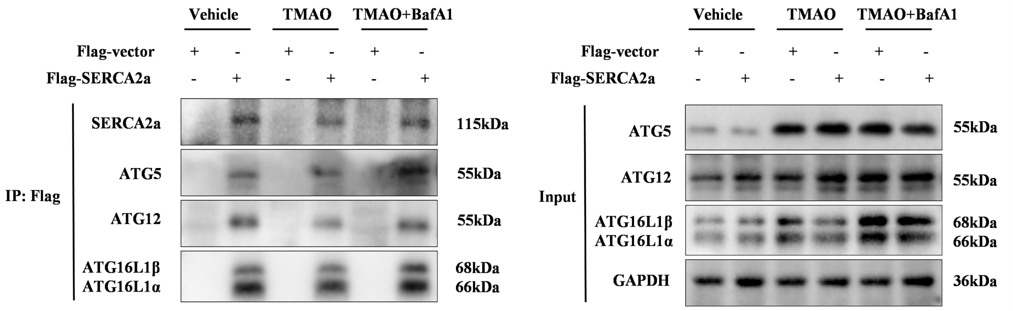

Figure. S9 SERCA2a-ATG5 interaction was enhanced under TMAO conditions after the addition of Bafilomycin A1, and by immunoprecipitation SERCA2a was further found to interact with the ATG5 complex (ATG12-ATG5-ATG16L1). H9c2 cells transfected with Flag-SERCA2a were cultured in the presence or absence of TMAO (1mM) for 48h, followed by treatment with BafA1(20nM) for 24h. Western blot analysis of ATG5, SERCA2a, ATG12 and ATG16L1.

Figure. S10:

The following are the results of all immunoblot replicates. Blot scans labelled as 'Repeat 1' match the figure panels presented in the manuscript. In figure 6b and 6g,we have used parallel gel (loading the same quantity of the protein to different gels) to run the western blot at the same time, as the molecular weight of the target protein and/or the control is relatively close.

Fig. 1 Blots

i

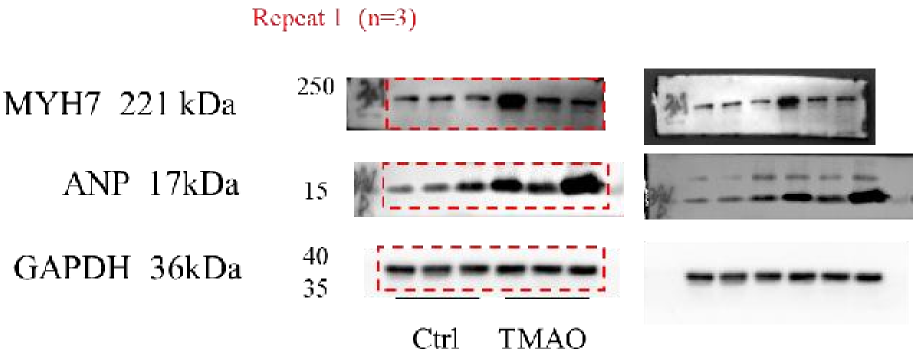

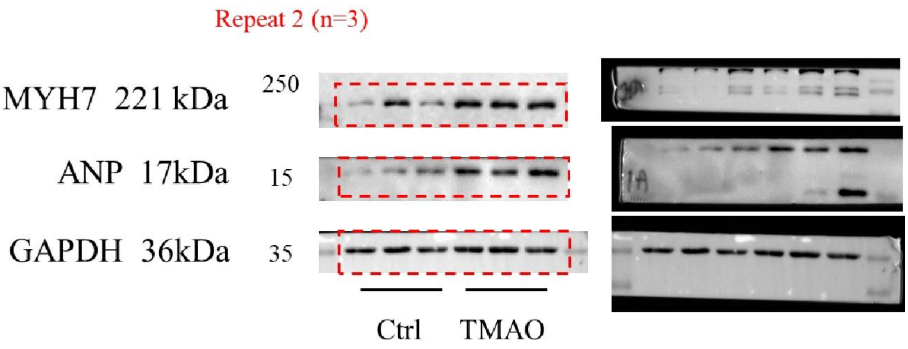

l

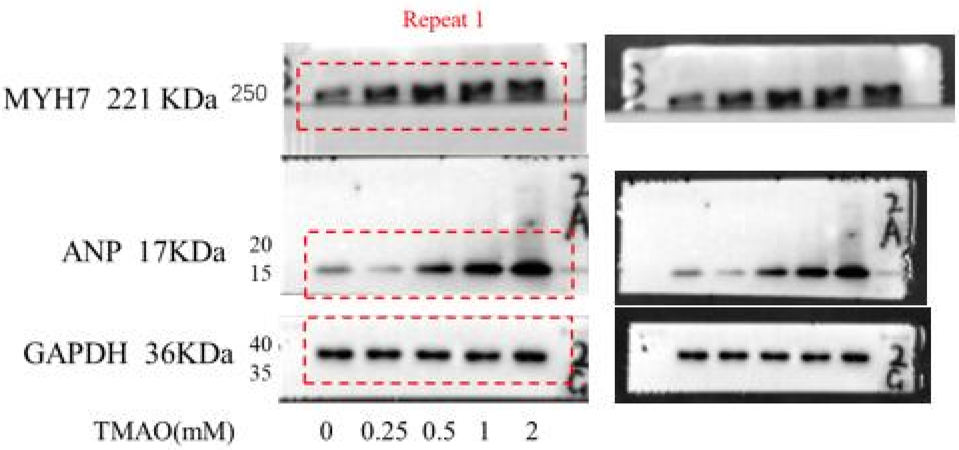

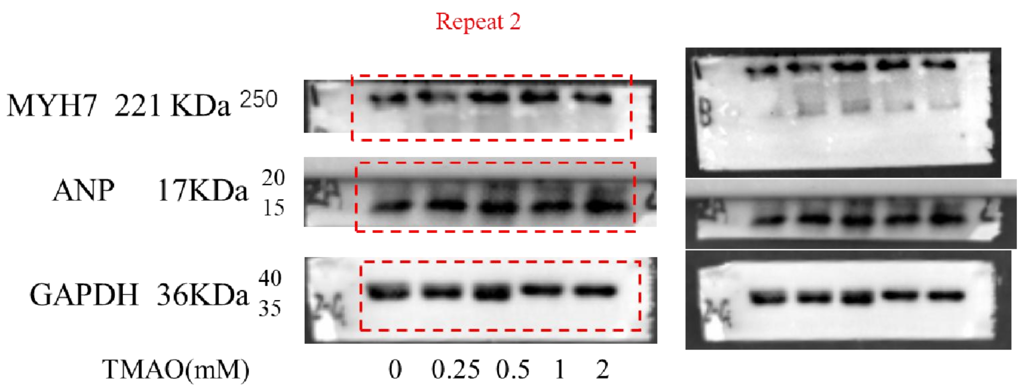
Repeat 2
MYH7 221 KDa 250
ANP 17KDa 20 15
GAPDH 36KDa 40 35
TMAO(mM) 0 0.25 0.5 1 2

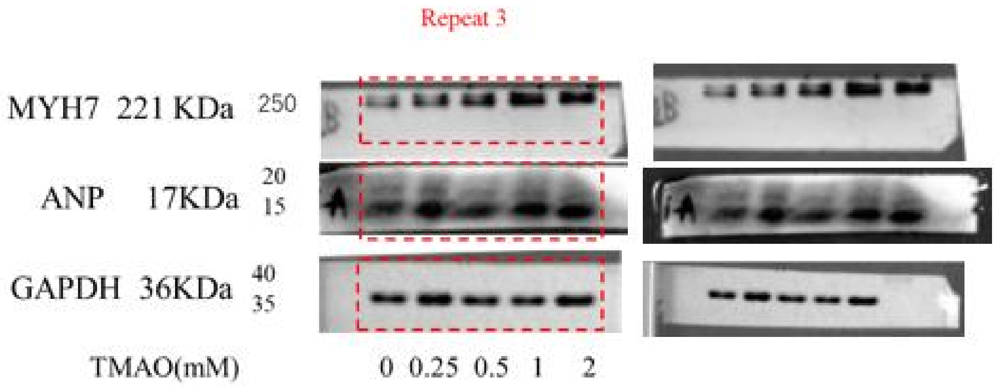
Repeat 3
MYH7 221 KDa 250
ANP 17KDa 20 15
GAPDH 36KDa 40 35
TMAO(mM) 0 0.25 0.5 1 2

o

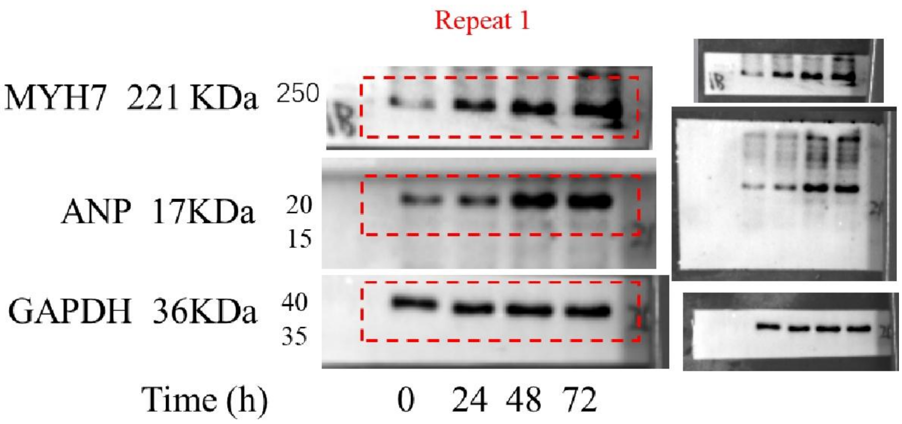
Repeat 1
MYH7 221 KDa 250
ANP 17KDa 20 15
GAPDH 36KDa 40 35
Time (h) 0 24 48 72

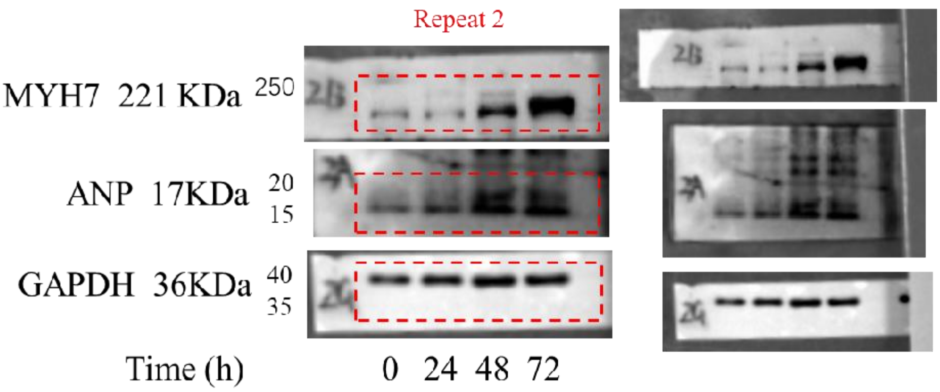
Repeat 2
MYH7 221 KDa 250
ANP 17KDa 20 15
GAPDH 36KDa 40 35
Time (h) 0 24 48 72

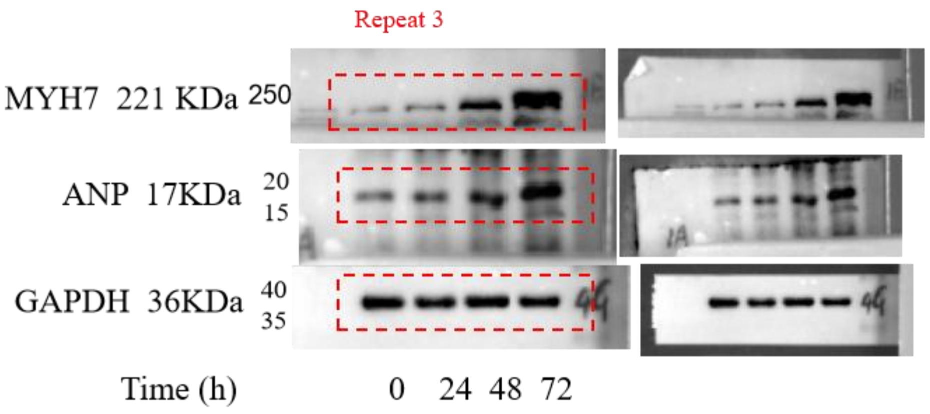

Fig. 2 Blots

d

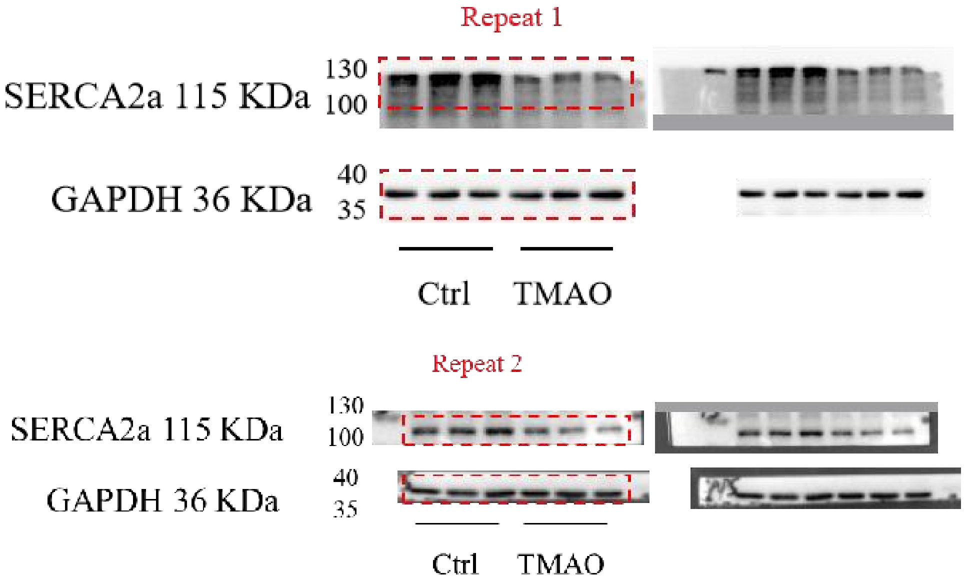

f

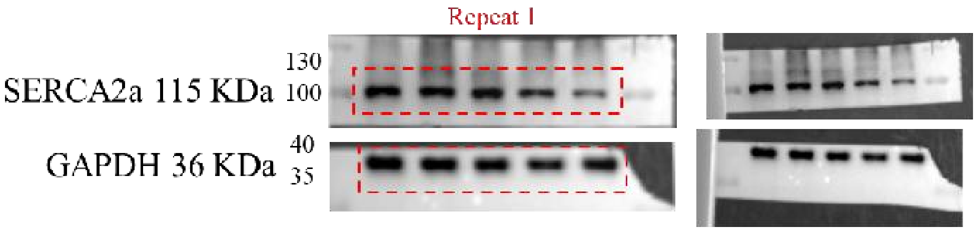

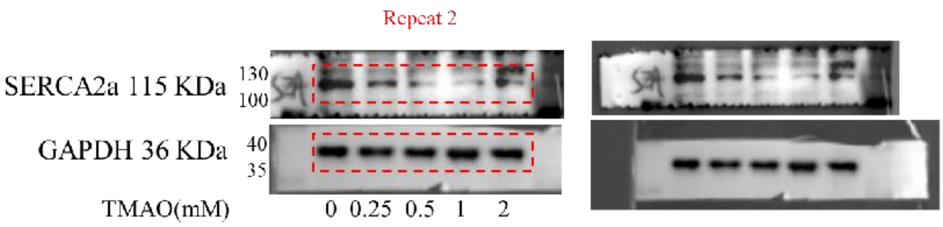
Repeat 2
SERCA2a 115 KDa
130
100
GAPDH 36 KDa
40
35
TMAO(mM)
0 0.25 0.5 1 2

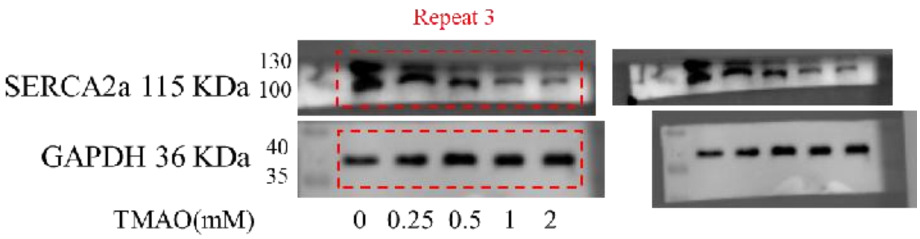
Repeat 3
SERCA2a 115 KDa
130
100
GAPDH 36 KDa
40
35
TMAO(mM)
0 0.25 0.5 1 2

h

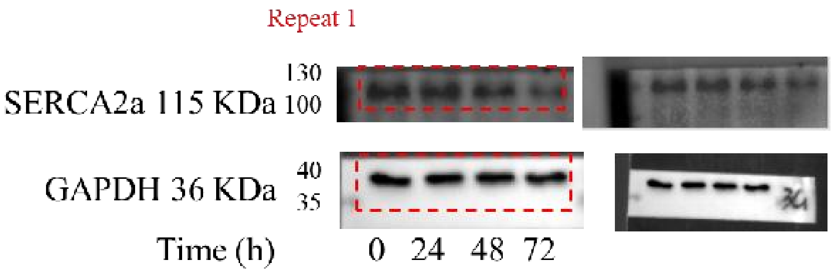
Repeat 1
SERCA2a 115 KDa
130
100
GAPDH 36 KDa
40
35
Time (h)
0 24 48 72

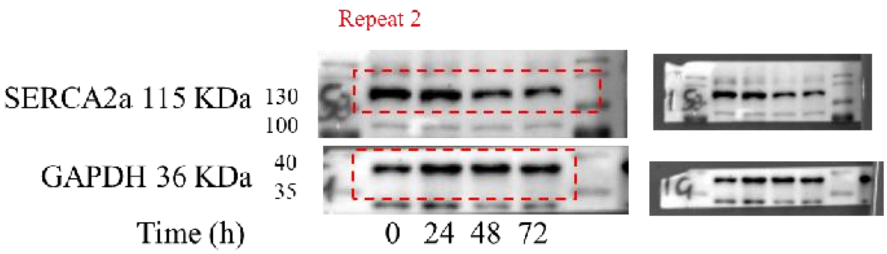
Repeat 2
SERCA2a 115 KDa
130
100
GAPDH 36 KDa
40
35
Time (h)
0 24 48 72

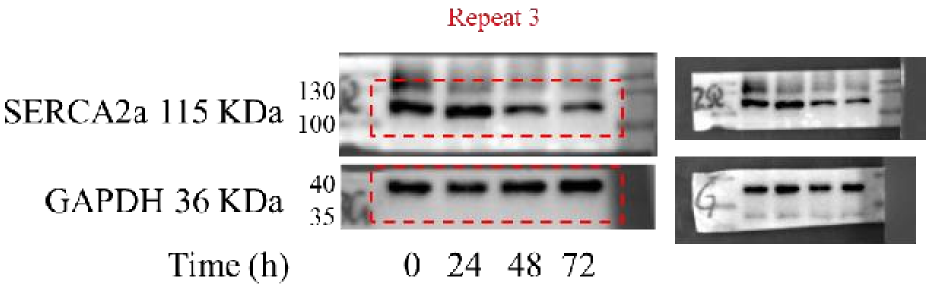
Repeat 3
SERCA2a 115 KDa
130
100
GAPDH 36 KDa
40
35
Time (h)
0 24 48 72

j

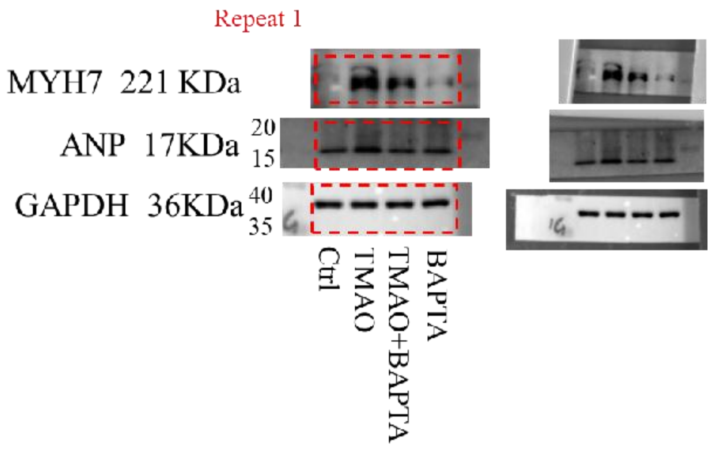
Repeat 1
MYH7 221 KDa
ANP 17KDa
20
15
GAPDH 36KDa
40
35
Ctrl
TMAO
TMAO+BAPTA
BAPTA

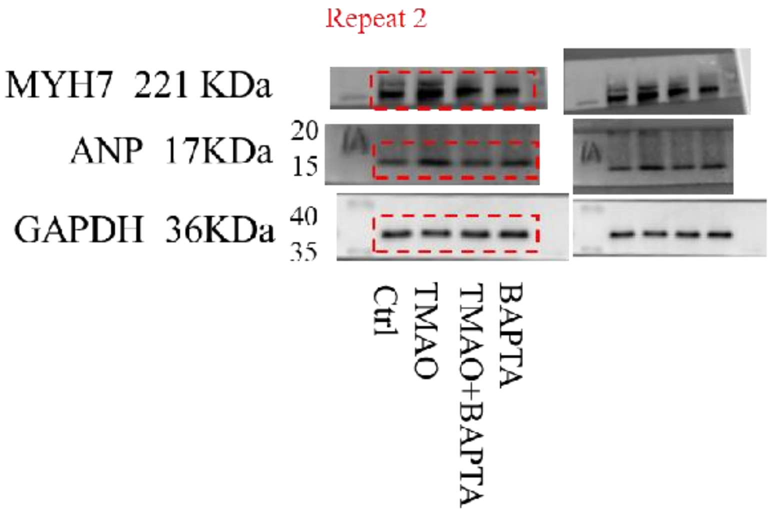
Repeat 2
MYH7 221 KDa
ANP 17KDa
20
15
GAPDH 36KDa
40
35
Ctrl
TMAO
TMAO+BAPTA
BAPTA

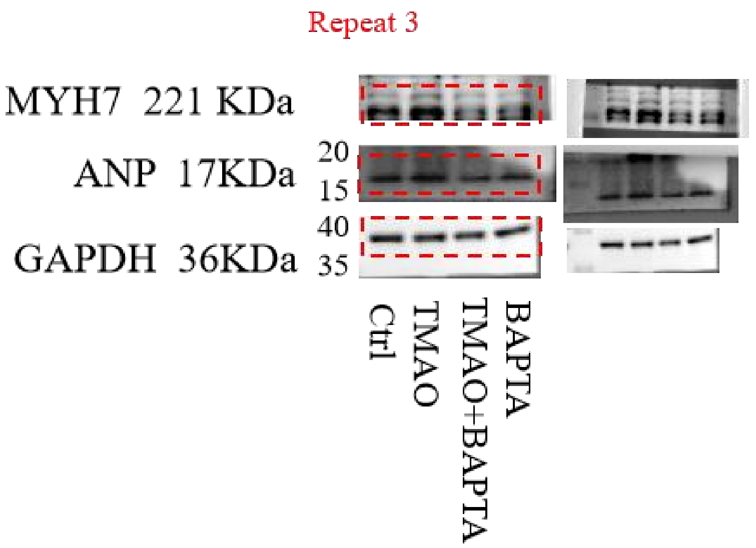
Repeat 3
MYH7 221 KDa
ANP 17KDa
20
15
GAPDH 36KDa
40
35
Ctrl
TMAO
TMAO+BAPTA
BAPTA

Fig. 3 Blot

f

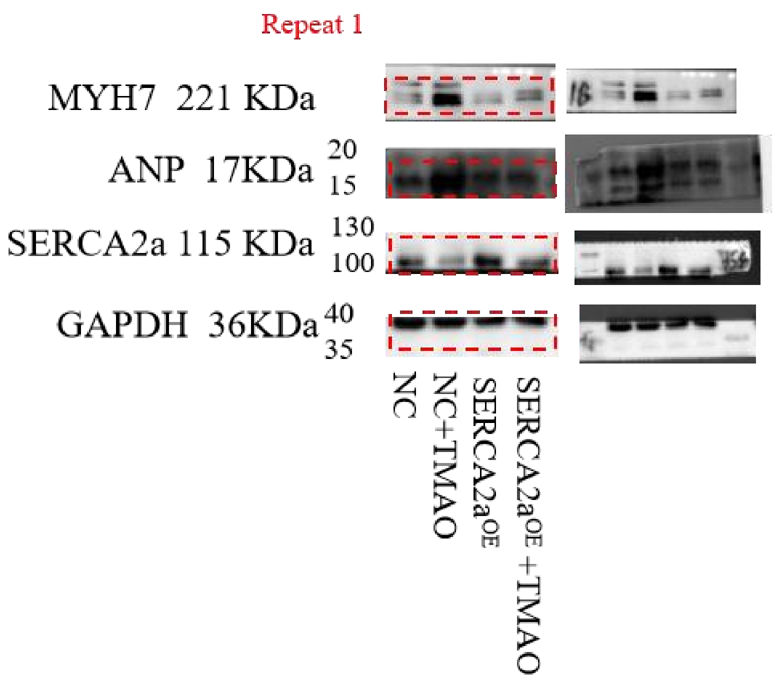
Repeat 1
MYH7 221 KDa
ANP 17KDa
20
15
SERCA2a 115 KDa
130
100
GAPDH 36KDa
40
35
NC
NC+TMAO
SERCA2a^OE
SERCA2a^OE +TMAO

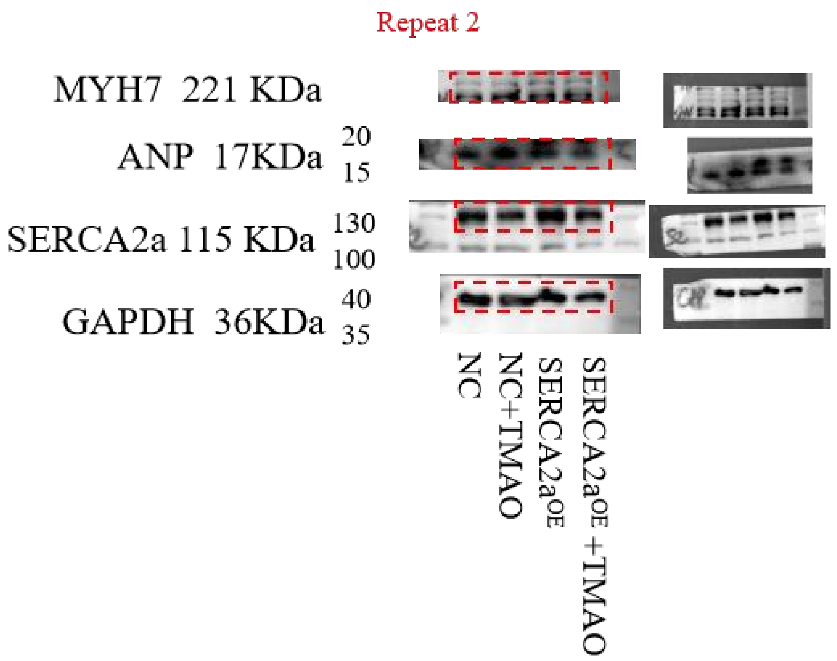
Repeat 2
MYH7 221 KDa
ANP 17KDa
20
15
SERCA2a 115 KDa
130
100
GAPDH 36KDa
40
35
NC
NC+TMAO
SERCA2a^OE
SERCA2a^OE +TMAO

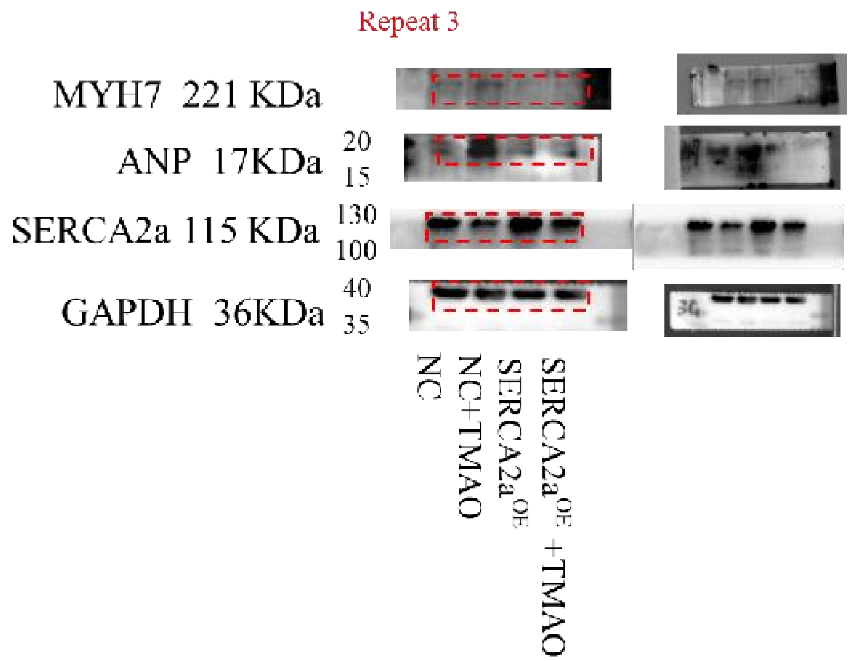

Repeat 3
MYH7 221 KDa
ANP 17KDa
20
15
SERCA2a 115 KDa
130
100
GAPDH 36KDa
40
35
NC
NC+TMAO
SERCA2a^{OE}
SERCA2a^{OE} +TMAO

Fig. 4 Blots

a

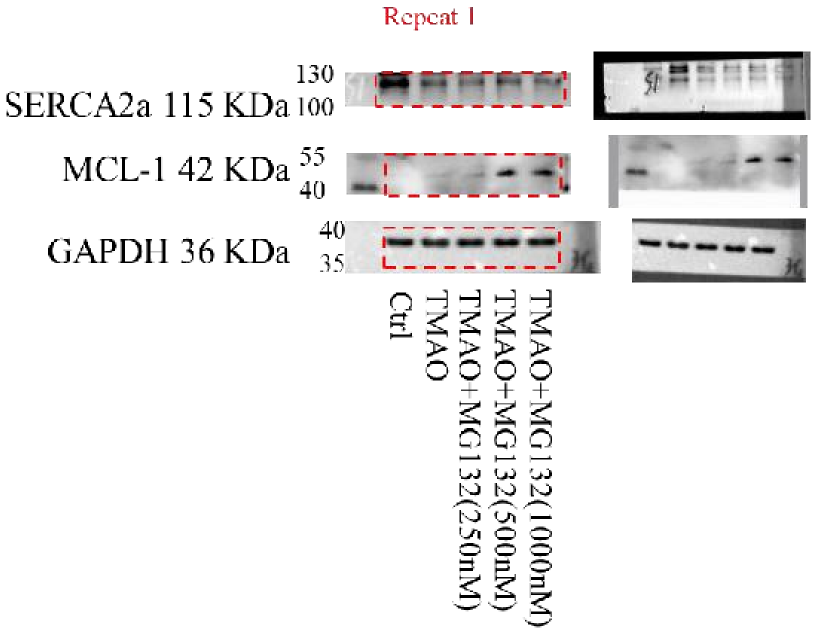

Repeat 1
SERCA2a 115 KDa
130
100
MCL-1 42 KDa
55
40
GAPDH 36 KDa
40
35
Ctrl
TMAO
TMAO+MG132(250nM)
TMAO+MG132(500nM)
TMAO+MG132(1000nM)

Repeat 2

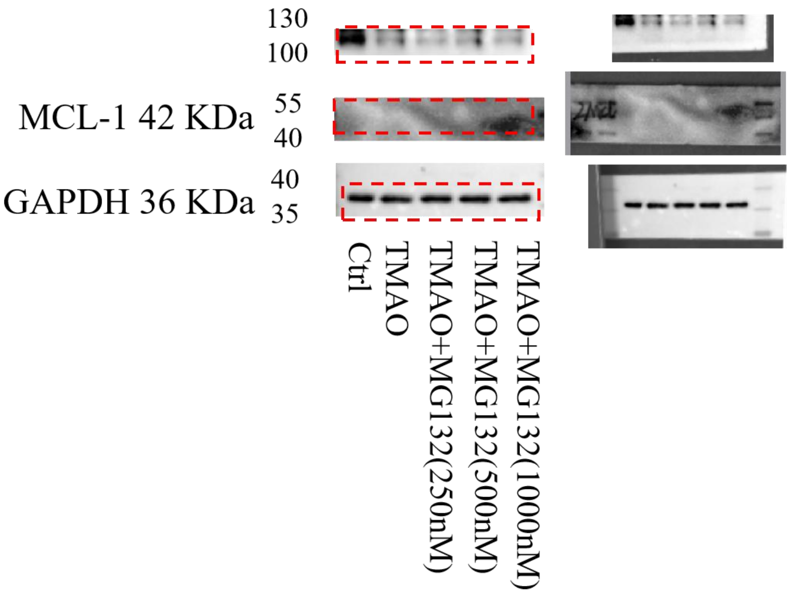

Repeat 3

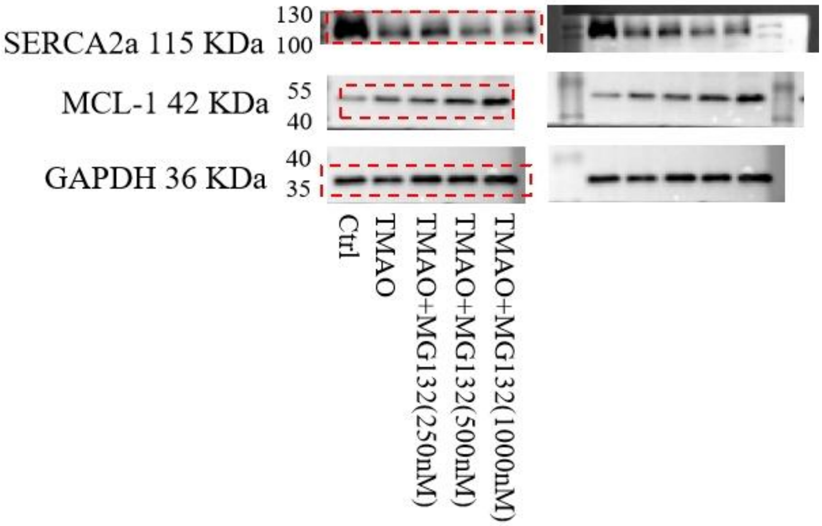

C

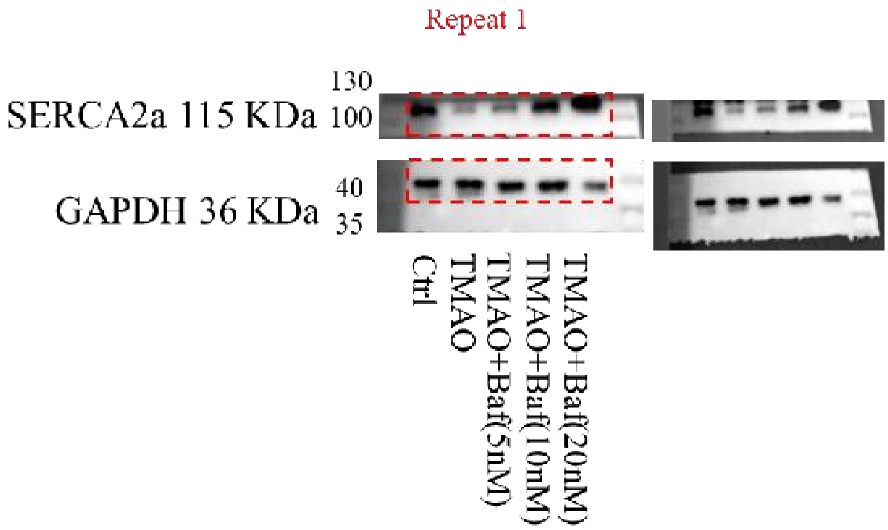
Repeat 1
SERCA2a 115 KDa
130
100
GAPDH 36 KDa
40
35
Ctrl
TMAO
TMAO+Baf(5nM)
TMAO+Baf(10nM)
TMAO+Baf(20nM)

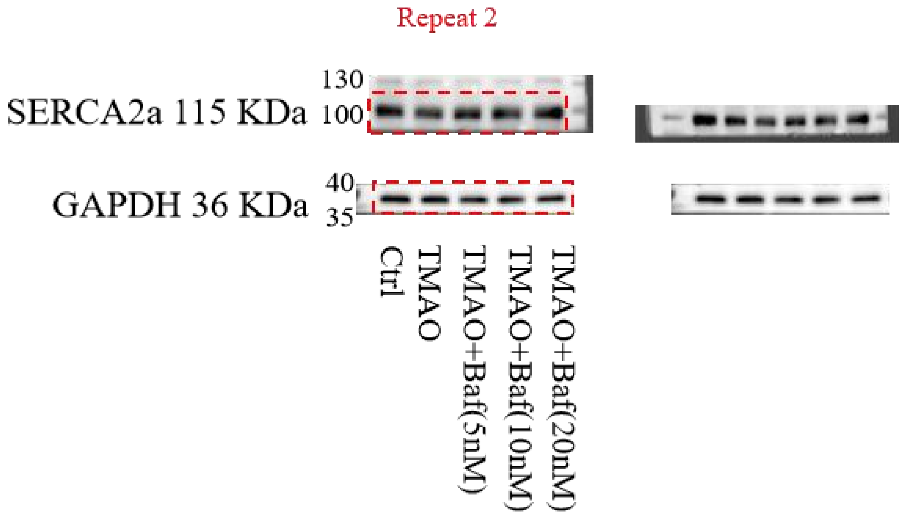
Repeat 2
SERCA2a 115 KDa
130
100
GAPDH 36 KDa
40
35
Ctrl
TMAO
TMAO+Baf(5nM)
TMAO+Baf(10nM)
TMAO+Baf(20nM)

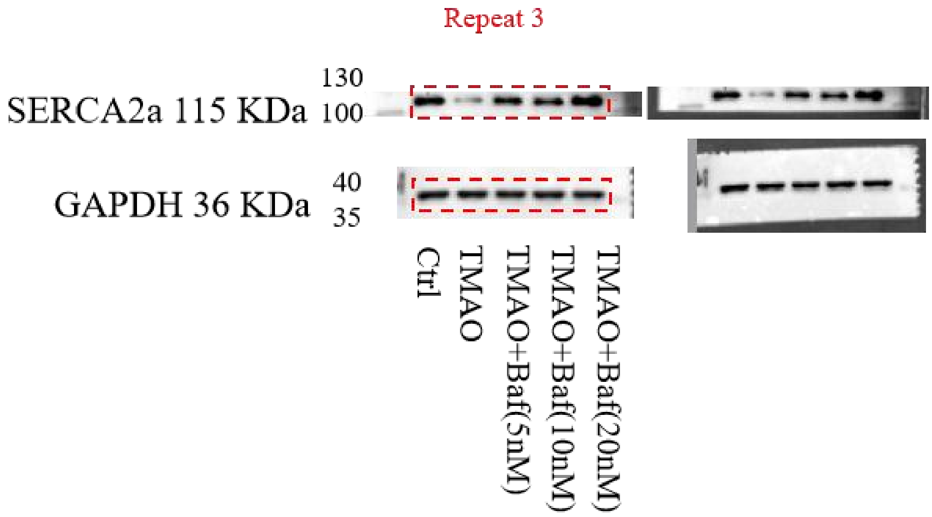
Repeat 3
SERCA2a 115 KDa
130
100
GAPDH 36 KDa
40
35
Ctrl
TMAO
TMAO+Baf(5nM)
TMAO+Baf(10nM)
TMAO+Baf(20nM)

e

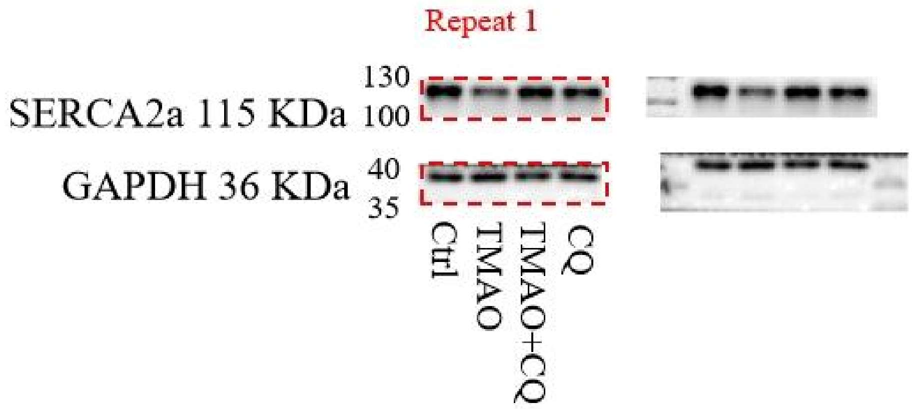
Repeat 1
130
SERCA2a 115 KDa
100
GAPDH 36 KDa
40
35
Ctrl
TMAO
TMAO+CQ
CQ

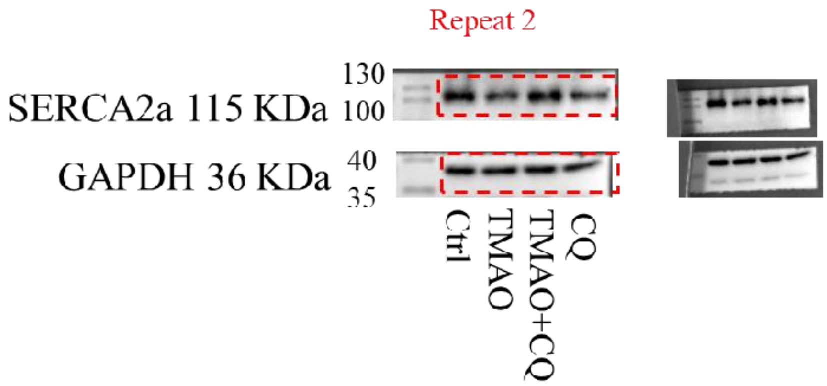
Repeat 2
130
SERCA2a 115 KDa
100
GAPDH 36 KDa
40
35
Ctrl
TMAO
TMAO+CQ
CQ

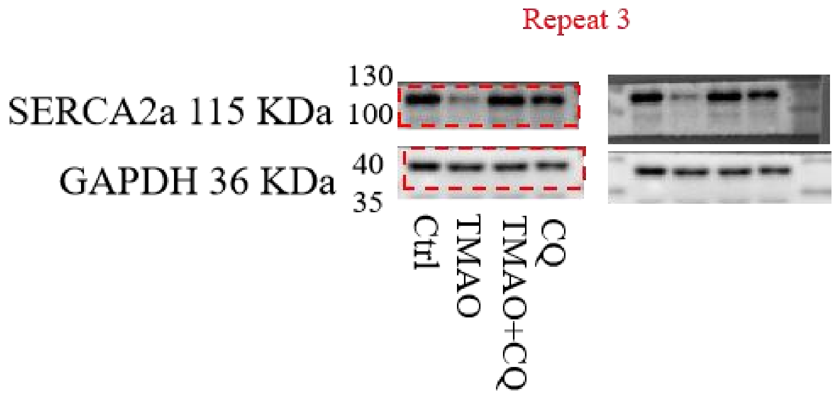
Repeat 3
130
SERCA2a 115 KDa
100
GAPDH 36 KDa
40
35
Ctrl
TMAO
TMAO+CQ
CQ

Fig. 5 Blot

b

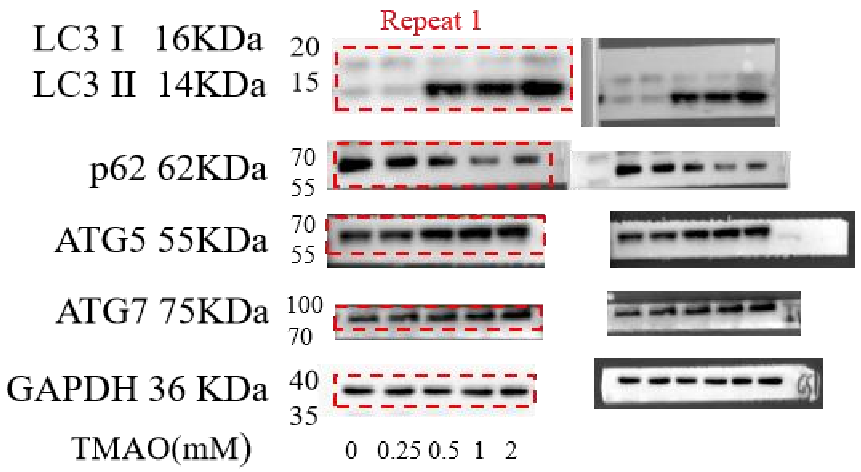
Repeat 1
LC3 I 16KDa
LC3 II 14KDa
20
15
p62 62KDa
70
55
ATG5 55KDa
70
55
ATG7 75KDa
100
70
GAPDH 36 KDa
40
35
TMAO(mM)
0 0.25 0.5 1 2

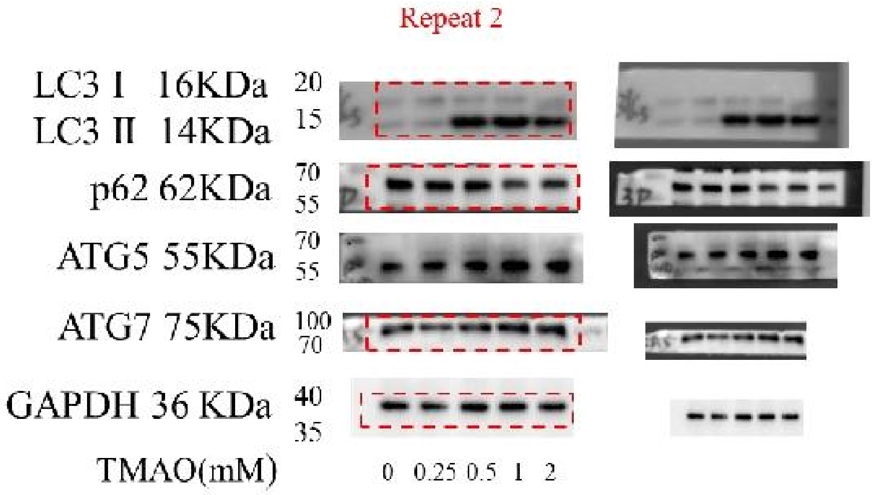
Repeat 2
LC3 I 16KDa
LC3 II 14KDa
20
15
p62 62KDa
70
55
ATG5 55KDa
70
55
ATG7 75KDa
100
70
GAPDH 36 KDa
40
35
TMAO(mM)
0 0.25 0.5 1 2

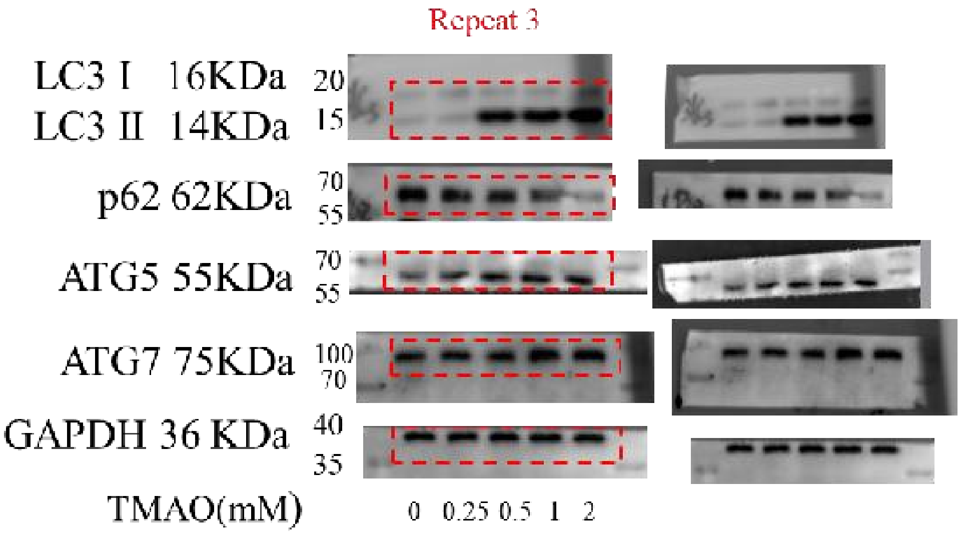
Repeat 3
LC3 I 16KDa
LC3 II 14KDa
20
15
p62 62KDa
70
55
ATG5 55KDa
70
55
ATG7 75KDa
100
70
GAPDH 36 KDa
40
35
TMAO(mM)
0 0.25 0.5 1 2

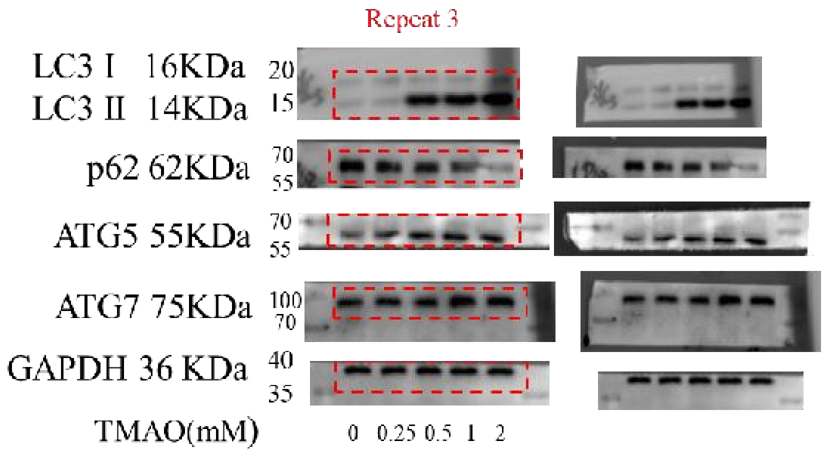
Repeat 3
LC3 I 16KDa
LC3 II 14KDa
20
15
p62 62KDa
70
55
ATG5 55KDa
70
55
ATG7 75KDa
100
70
GAPDH 36 KDa
40
35
TMAO(mM)
0 0.25 0.5 1 2

g

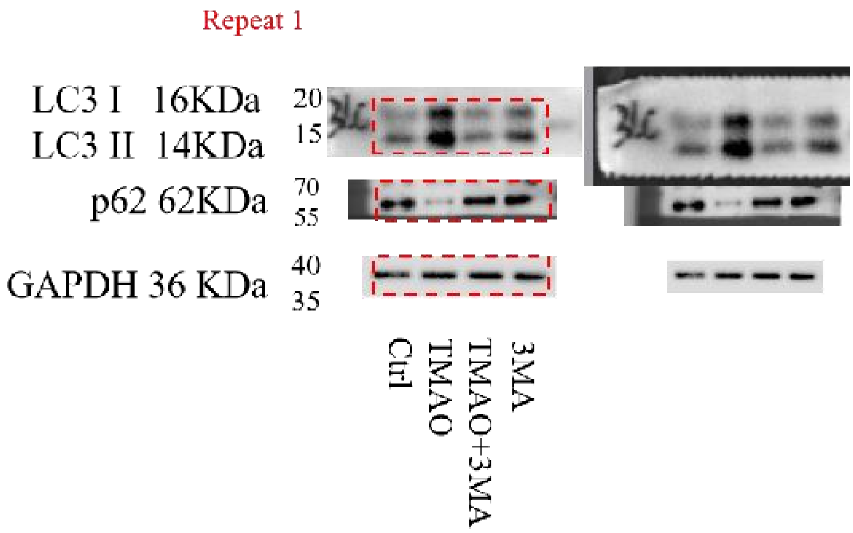
Repeat 1
LC3 I 16KDa
LC3 II 14KDa
20
15
p62 62KDa
70
55
GAPDH 36 KDa
40
35
Ctrl
TMAO
TMAO+3MA
3MA

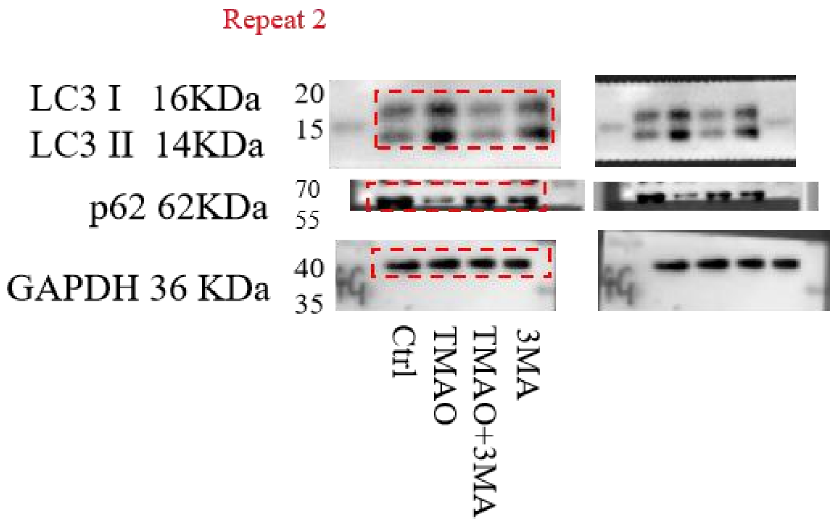
Repeat 2
LC3 I 16KDa
LC3 II 14KDa
20
15
p62 62KDa
70
55
GAPDH 36 KDa
40
35
Ctrl
TMAO
TMAO+3MA
3MA

Repeat 3

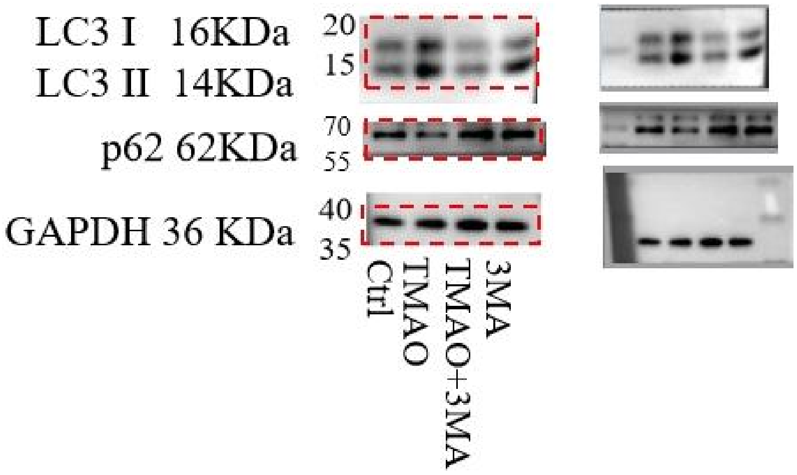

q

Repeat 1

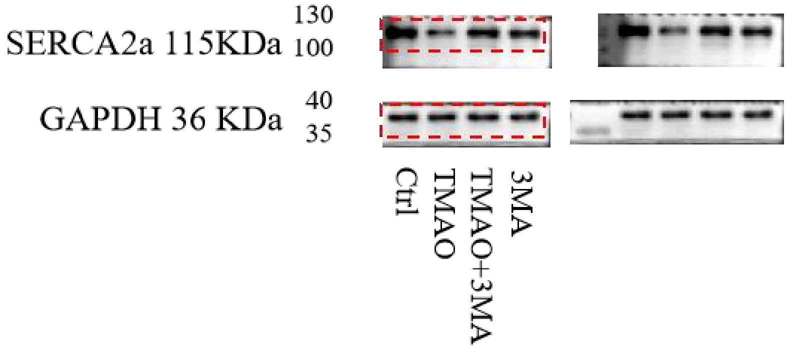

Repeat 2

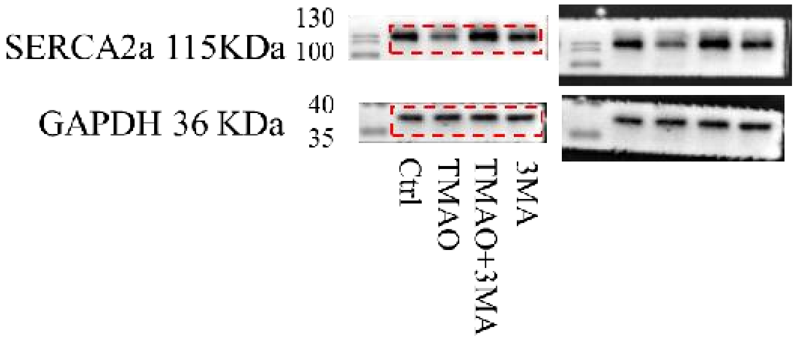

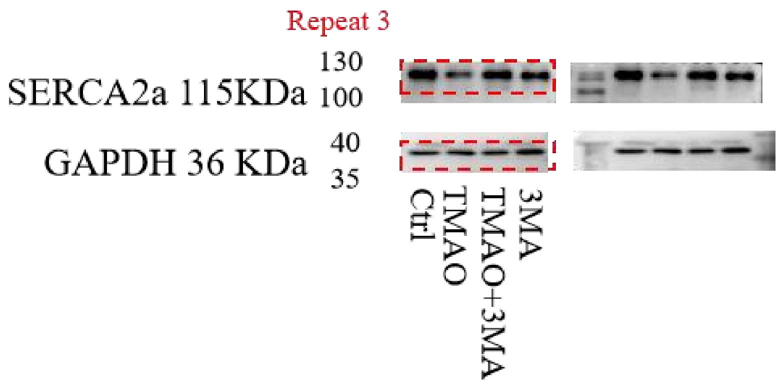
Repeat 3
SERCA2a 115KDa
130
100
GAPDH 36 KDa
40
35
Ctrl
TMAO
TMAO+3MA
3MA

s

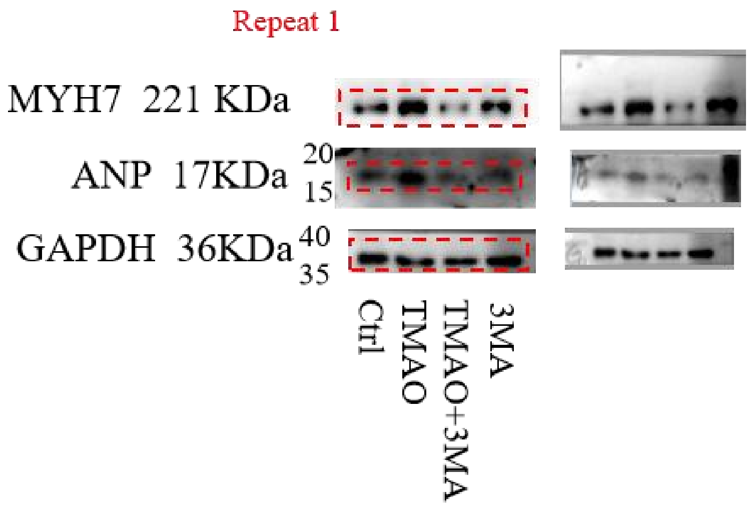
Repeat 1
MYH7 221 KDa
ANP 17KDa
20
15
GAPDH 36KDa
40
35
Ctrl
TMAO
TMAO+3MA
3MA

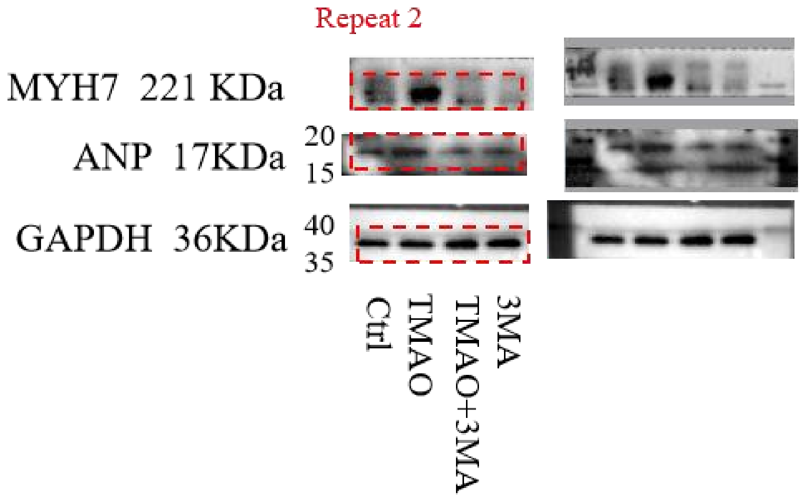
Repeat 2
MYH7 221 KDa
ANP 17KDa
20
15
GAPDH 36KDa
40
35
Ctrl
TMAO
TMAO+3MA
3MA

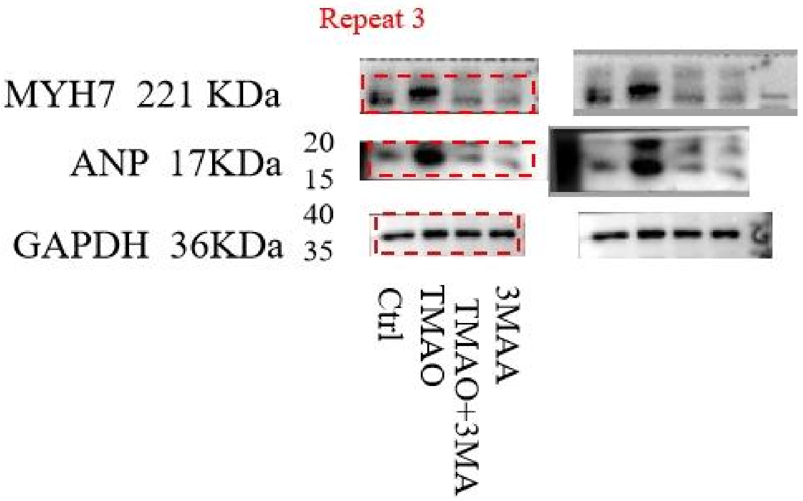
Repeat 3
MYH7 221 KDa
ANP 17KDa
20
15
GAPDH 36KDa
40
35
Ctrl
TMAO
TMAO+3MA
3MAA

Fig. 6 Blot

b

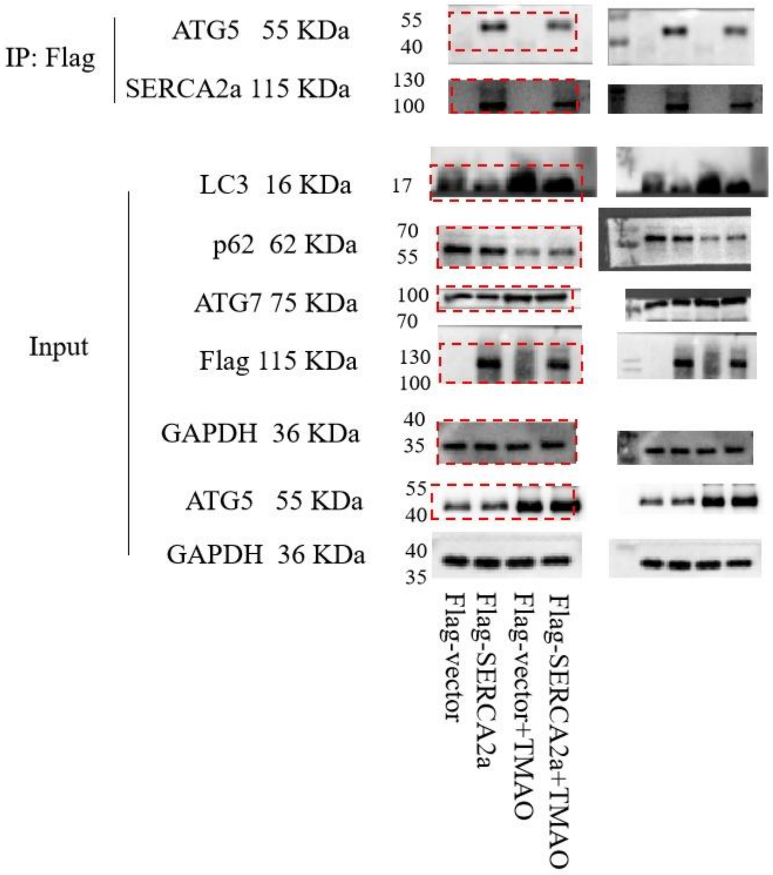
IP: Flag
ATG5 55 KDa
SERCA2a 115 KDa
55
40
130
100
Input
LC3 16 KDa
17
p62 62 KDa
70
55
ATG7 75 KDa
100
70
Flag 115 KDa
130
100
GAPDH 36 KDa
40
35
ATG5 55 KDa
55
40
GAPDH 36 KDa
40
35
Flag-vector
Flag-SERCA2a
Flag-vector+TMAO
Flag-SERCA2a+TMAO

g

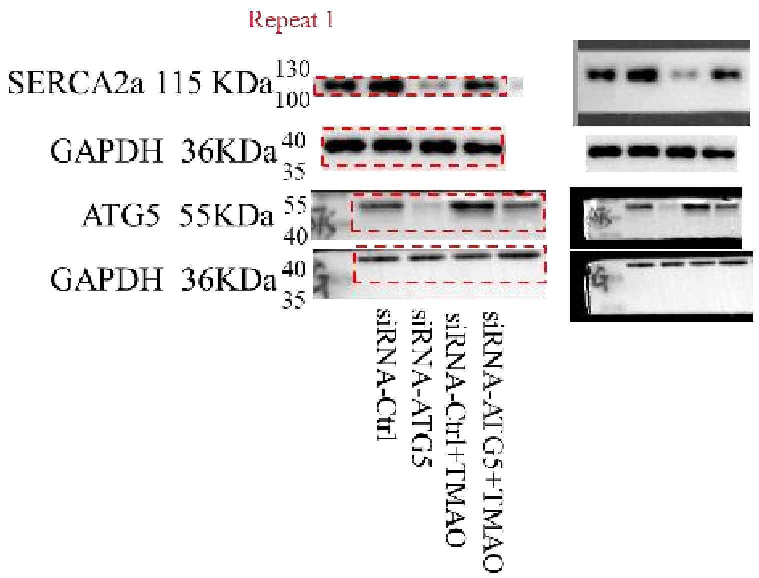
Repeat 1
SERCA2a 115 KDa
130
100
GAPDH 36KDa
40
35
ATG5 55KDa
55
40
GAPDH 36KDa
40
35
siRNA-Ctrl
siRNA-ATG5
siRNA-Ctrl+TMAO
siRNA-ATG5+TMAO

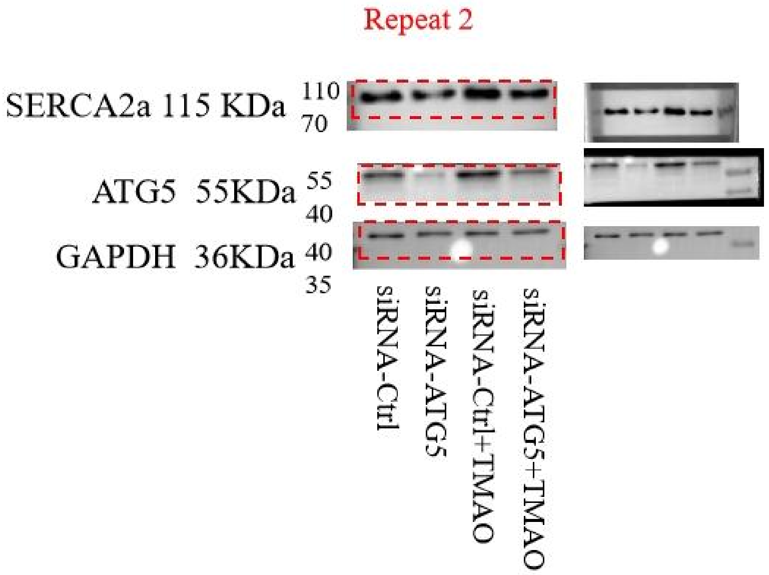
Repeat 2
SERCA2a 115 KDa
110
70
ATG5 55KDa
55
40
GAPDH 36KDa
40
35
siRNA-Ctrl
siRNA-ATG5
siRNA-Ctrl+TMAO
siRNA-ATG5+TMAO

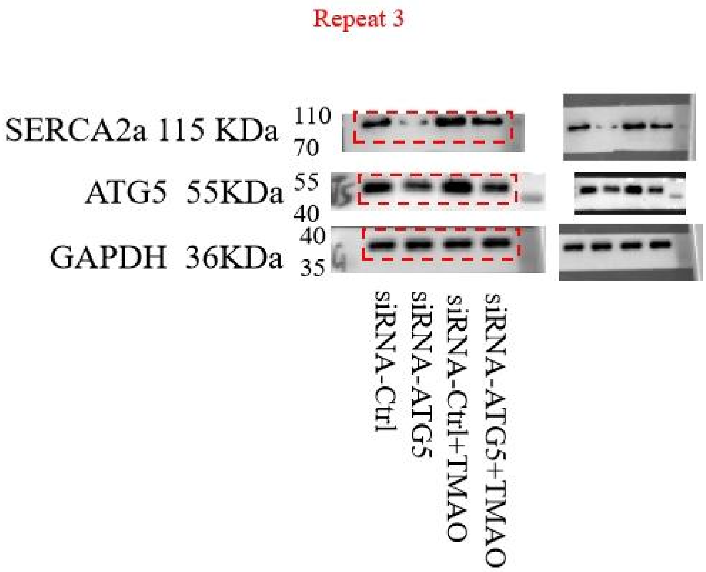
Repeat 3
SERCA2a 115 KDa
110
70
ATG5 55KDa
55
40
GAPDH 36KDa
40
35
siRNA-Ctrl
siRNA-ATG5
siRNA-Ctrl+TMAO
siRNA-ATG5+TMAO

j

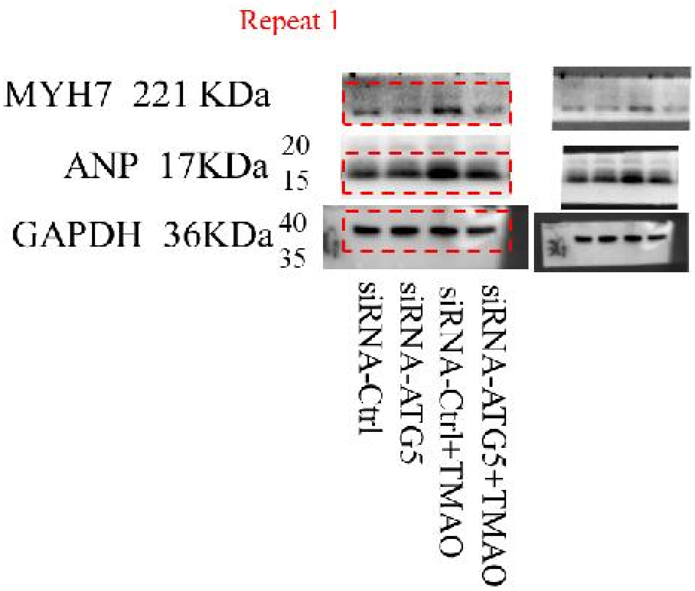
Repeat 1
MYH7 221 KDa
ANP 17KDa
20
15
GAPDH 36KDa
40
35
siRNA-Ctrl
siRNA-ATG5
siRNA-Ctrl+TMAO
siRNA-ATG5+TMAO

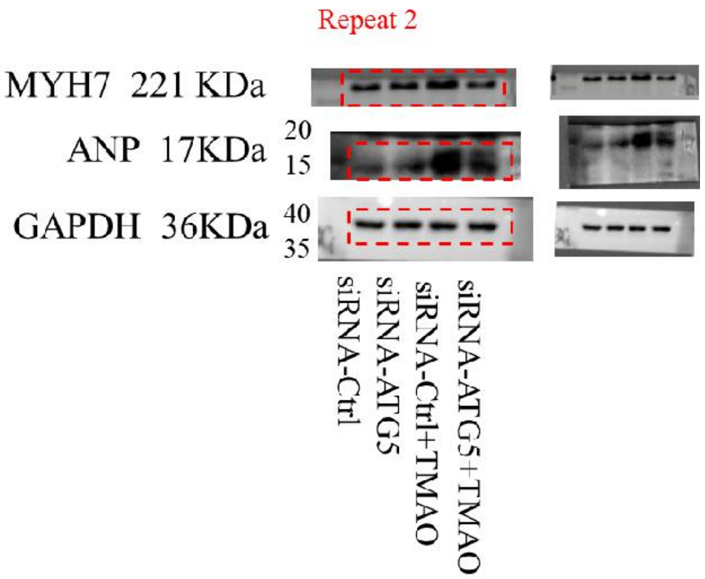
Repeat 2
MYH7 221 KDa
ANP 17KDa
20
15
GAPDH 36KDa
40
35
siRNA-Ctrl
siRNA-ATG5
siRNA-Ctrl+TMAO
siRNA-ATG5+TMAO

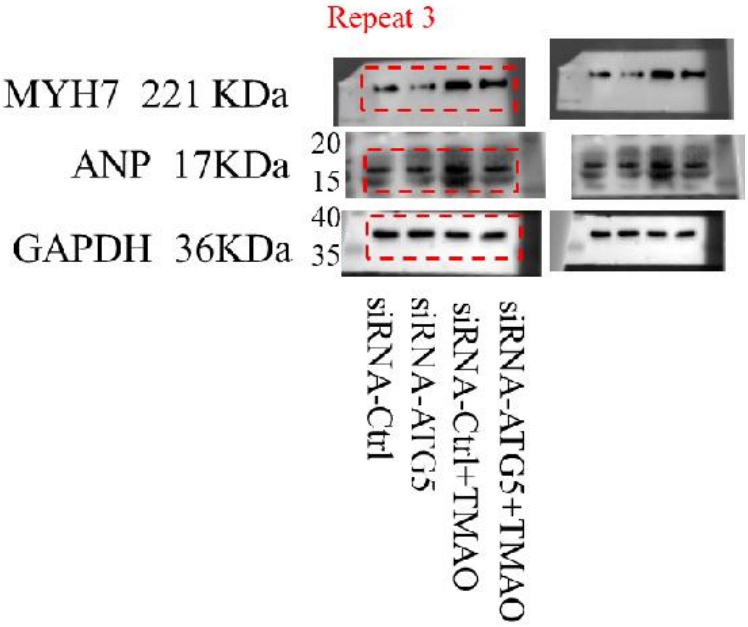
Repeat 3
MYH7 221 KDa
ANP 17KDa
20
15
GAPDH 36KDa
40
35
siRNA-Ctrl
siRNA-ATG5
siRNA-Ctrl+TMAO
siRNA-ATG5+TMAO

Fig. 7 Blot
i

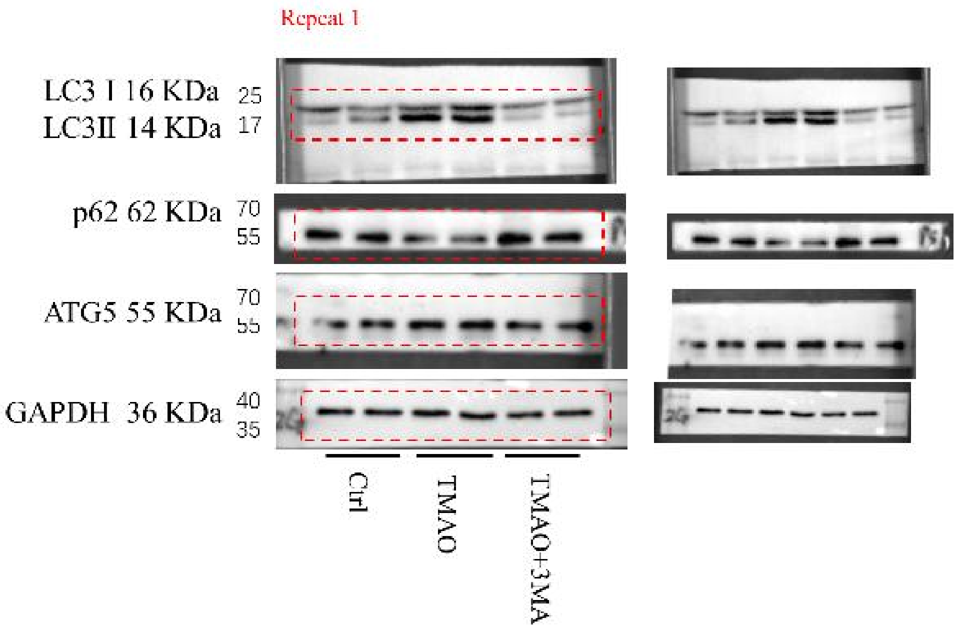
Repeat 1
LC3 I 16 KDa
LC3II 14 KDa
25
17
p62 62 KDa
70
55
ATG5 55 KDa
70
55
GAPDH 36 KDa
40
35
Ctrl
TMAO
TMAO+3MA

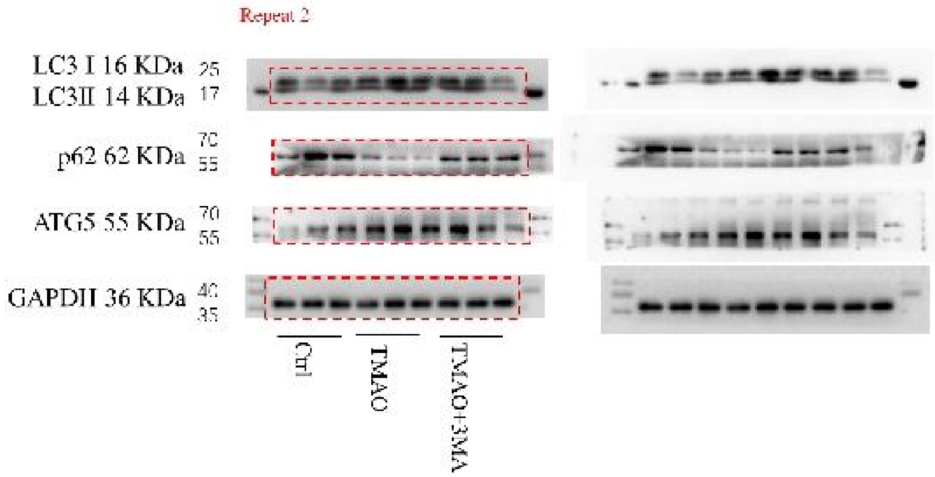
Repeat 2
LC3 I 16 KDa
LC3II 14 KDa
25
17
p62 62 KDa
70
55
ATG5 55 KDa
70
55
GAPDH 36 KDa
40
35
Ctrl
TMAO
TMAO+3MA

m

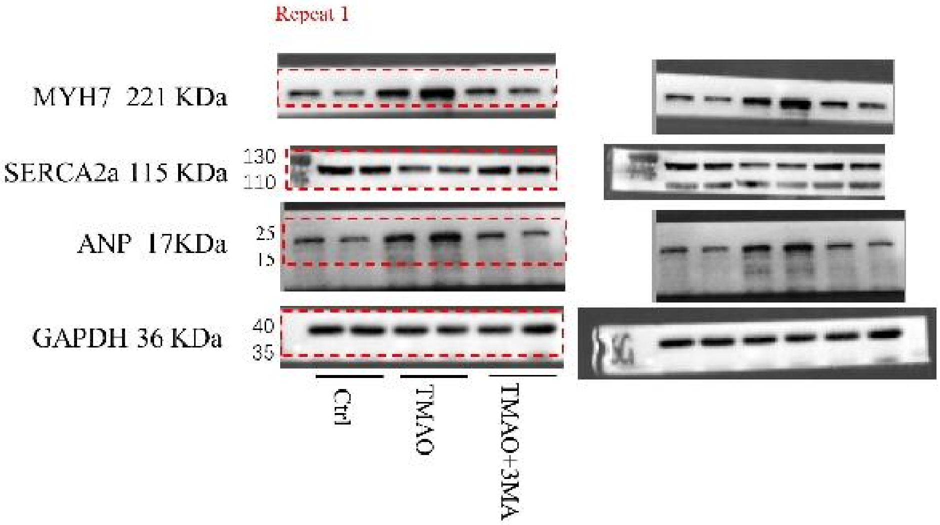
Repeat 1
MYH7 221 KDa
SERCA2a 115 KDa
130
110
ANP 17KDa
25
15
GAPDH 36 KDa
40
35
Ctrl
TMAO
TMAO+3MA

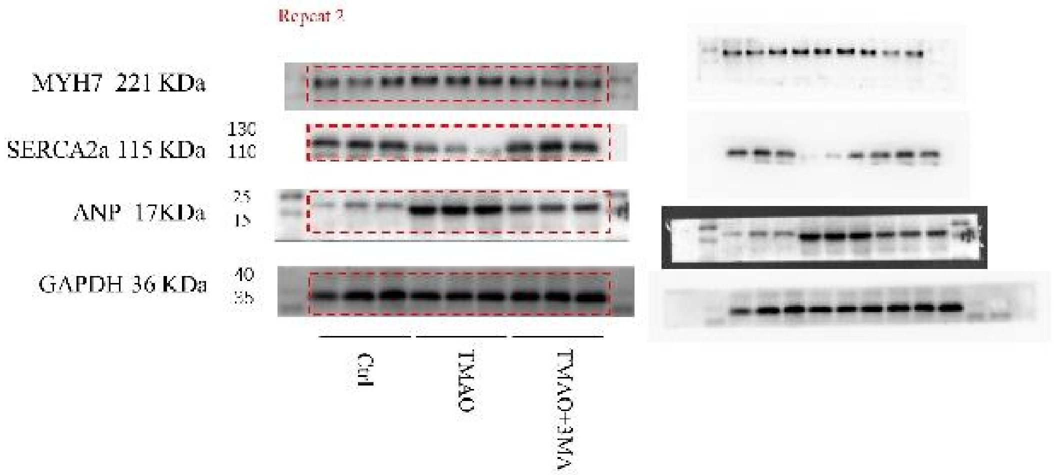

Fig. S1 Blot

i

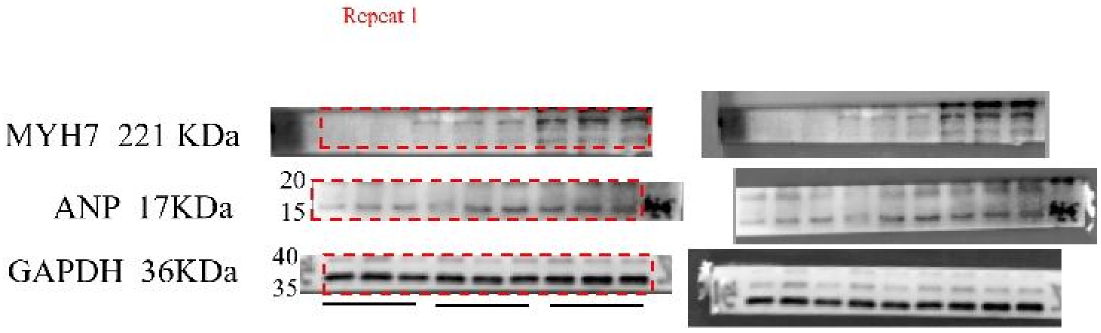

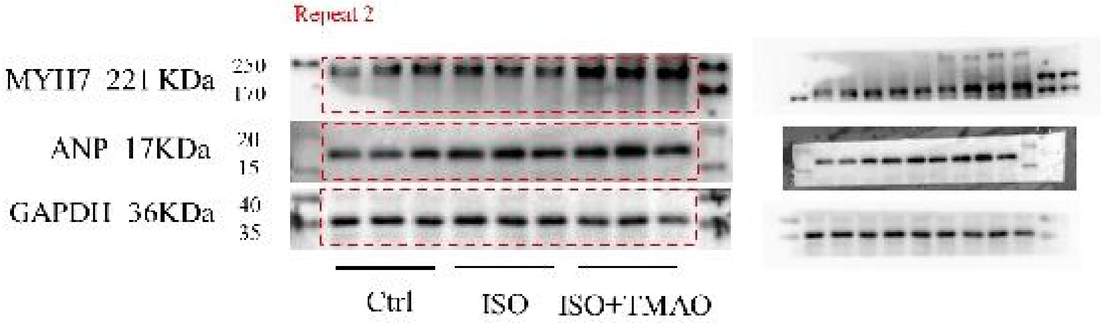

Fig. S2 Blot

i

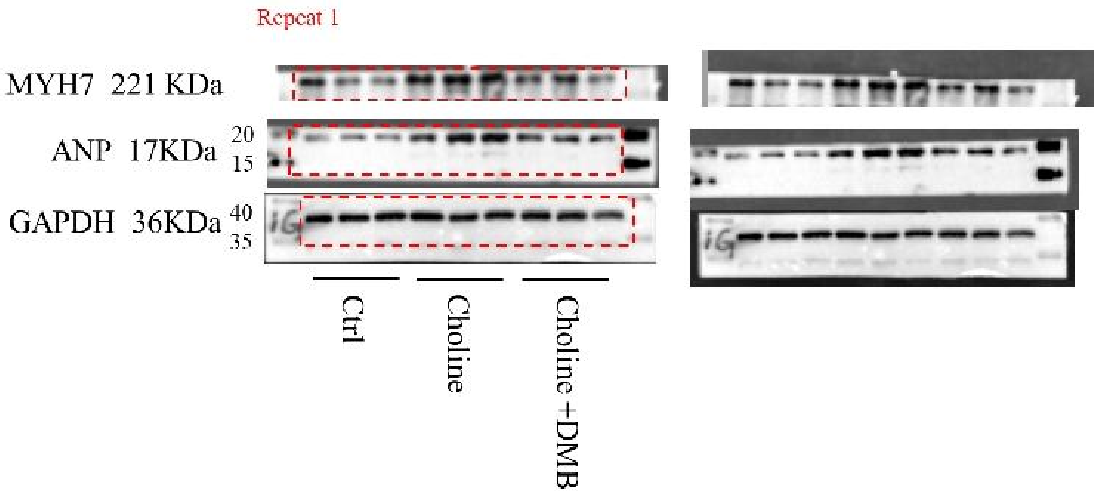
Repeat 1
MYH7 221 KDa
ANP 17KDa
20
15
GAPDH 36KDa
40
35
Ctrl
Choline
Choline +DMB

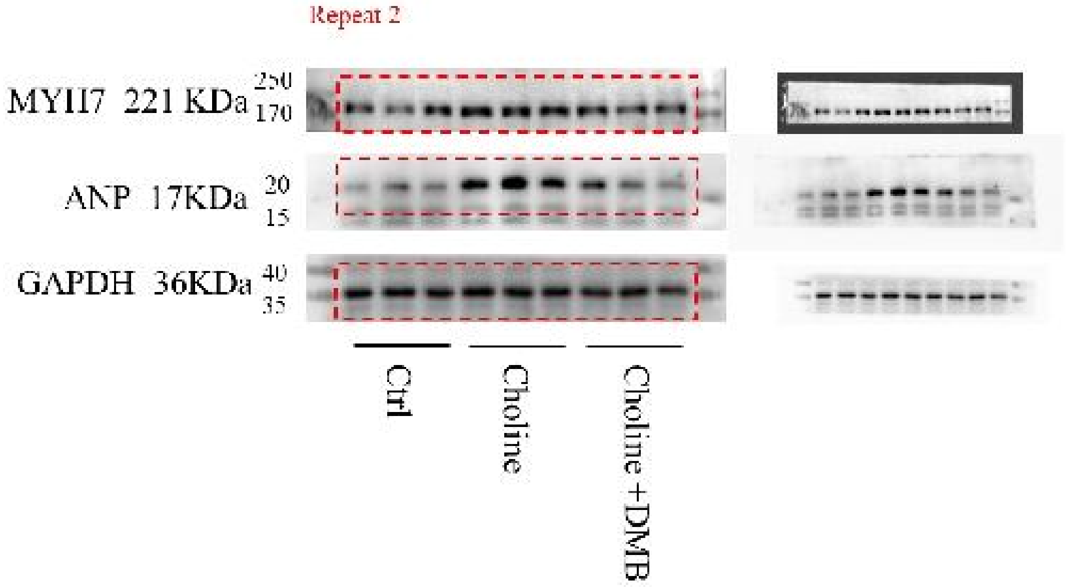
Repeat 2
MYH7 221 KDa
250
170
ANP 17KDa
20
15
GAPDH 36KDa
40
35
Ctrl
Choline
Choline +DMB
