# Supplementary material for: The gut microbiota metabolite trimethylamine N-oxide promotes cardiac hypertrophy by activating the autophagic degradation of SERCA2a
Source: Commun Biol. 2025 Apr 10;8:596. doi: 10.1038/s42003-025-08016-9 (PMC11986001; doi:10.1038/s42003-025-08016-9)
Supplement: Supplementary file 4 — Supplementary Data 1 [file 42003_2025_8016_MOESM4_ESM.pdf]

**Figure 1**							
**b**		c		**e**		**f**	
Control	TMAO	Control	TMAO	Control	TMAO	Control	TMAO
71.00	54.00	34.00	23.00	6.32	8.19	4.35	5.60
78.00	50.00	41.00	27.00	5.99	8.06	4.35	5.71
71.00	52.00	35.00	30.00	5.70	7.66	4.30	5.40
65.00	54.00	30.00	23.00	6.00	8.42	4.70	5.59
				7.11	8.39	4.46	5.51
				7.06	7.80	4.19	5.69
				6.91	8.00	4.45	5.62
				7.04	8.20	4.46	5.20
**g**		j		**k**			
Control	TMAO	Control	TMAO	Control	TMAO		
20.96	362.07	0.54	3.03	0.12	1.21		
12.31	362.07	0.45	1.36	0.16	1.45		
8.05	222.09	0.45	1.06	0.14	1.21		
10.38	122.55	0.60	1.20	0.54	1.00		
12.60	358.16	0.55	1.00	0.35	2.00		
15.13	287.60	0.30	2.02	0.23	1.00		
12.44	300.07						
12.78	400.07						
**m**							
0mM	0.25mM	0.5mM	1mM	2mM			
0.54	0.78	0.95	1.02	0.99			
0.50	1.23	1.45	1.48	1.61			
0.25	0.82	1.18	1.24	1.54			
**n**							
0mM	0.25mM	0.5mM	1mM	2mM			
0.20	0.37	0.51	0.99	1.21			
0.16	0.19	0.90	1.18	1.39			
0.32	0.46	0.61	0.78	0.86			
**p**							
0h	24h	48h	72h				
0.44	0.63	1.14	0.98				
0.32	0.70	0.87	1.47				
0.30	0.64	0.95	1.29				
**q**					**s**		
0h	24h	48h	72h		Control	TMAO	
0.45	0.64	1.31	1.35		1.42	2.31	
0.20	0.47	1.01	1.24		0.98	3.44	
0.11	0.58	1.42	1.49		1.01	5.84	
					0.60	4.39	
**Figure 2**							
**b**							
0	0.25mM	0.5mM	1mM	2mM			
1.00	1.07	1.18	1.16	1.27			
1.00	1.08	1.10	1.16	1.22			
1.00	1.12	1.14	1.11	1.27			
1.00	1.00	0.91	1.36	1.29			
c						e	
T(time)	0mM	0.25mM	0.5mM	1mM	2mM	Ctrl	TMAO
0s	1	1.000	1.093	0.990	1.124	1.24	0.41
	1	1.168	1.077	1.120	1.174	1.35	0.35
	1	0.979	1.012	0.919	1.008	1.45	0.21
	1	0.935	1.065	0.906	0.869	1.14	0.51
	1	0.907	1.010	0.855	0.959	1.25	0.35

18s	1	0.917	0.980	0.944	1.036	1.05	0.41
	1	1.081	1.052	1.123	1.046		
	1	0.978	0.987	0.989	0.992		
	1	0.997	1.089	0.946	0.876		
	1	0.947	0.982	0.896	0.920		
36s	1	0.912	1.078	0.970	1.027		
	1	1.024	0.986	1.116	1.032		
	1	0.939	0.936	0.980	1.071		
	1	0.935	1.076	0.885	0.912		
	1	1.001	1.055	0.953	0.970		
54s	1	0.950	1.036	0.952	1.074		
	1	1.017	0.992	1.071	1.062		
	1	1.058	1.031	1.056	1.147		
	1	0.913	1.014	0.866	0.801		
	1	0.873	1.035	0.903	0.963		
72s	1	1.210	1.260	1.450	1.515		
	1	1.228	1.150	1.025	1.447		
	1	1.100	1.072	0.972	1.368		
		1.101	1.270	1.159	1.218		
	1	1.093	1.226	1.257	1.376		
90s	1	1.110	1.165	1.443	1.406		
	1	1.194	1.126	1.030	1.326		
	1	1.123	1.125	1.047	1.457		
	1	1.168	1.273	1.256	1.340		
	1	1.129	1.272	1.274	1.402		
108s	1	1.080	1.166	1.373	1.418		
	1	1.205	1.210	1.344	1.471		
	1	1.228	1.205	1.283	1.456		
	1	1.199	1.304	1.288	1.324		
	1	1.122	1.274	1.232	1.330		
126s	1	1.060	1.079	1.362	1.387		
	1	1.178	1.192	1.342	1.504		
	1	1.187	1.120	1.290	1.403		
	1	1.136	1.219	1.233	1.295		
	1	1.152	1.217	1.225	1.348		
144s	1	1.130	1.211	1.376	1.455		
	1	1.152	1.116	1.262	1.440		
	1	1.149	1.044	1.275	1.448		
	1	1.120	1.261	1.210	1.220		
	1	1.105	1.259	1.281	1.390		
162s	1	1.120	1.267	1.401	1.433		
	1	1.246	1.255	1.338	1.470		
	1	1.206	1.181	1.383	1.557		
	1	1.073	1.192	1.125	1.196		
	1	1.041	1.200	1.210	1.312		
180s	1	1.160	1.212	1.377	1.474		
	1	1.181	1.149	1.321	1.444		
	1	1.179	1.131	1.292	1.452		
	1	1.058	1.150	1.208	1.215		
	1	1.062	1.169	1.169	1.267		
198s	1	1.110	1.174	1.333	1.475		
	1	1.121	1.183	1.252	1.455		
	1	1.216	1.035	1.307	1.446		
	1	1.220	1.279	1.327	1.304		
	1	1.115	1.218	1.194	1.374		
216s	1	1.150	1.229	1.435	1.515		
	1	1.259	1.221	1.310	1.480		
		1.174	1.180	1.455	1.508		
		1.103	1.155	1.178	1.177		
	1	1.056	1.153	1.220	1.300		
234s	1	1.150	1.289	1.399	1.472		
	1	1.148	1.156	1.216	1.381		
	1	1.180	1.100	1.337	1.497		
	1	1.120	1.159	1.239	1.280		

	1	1.226	1.263	1.304	1.443		
252s	1	1.110	1.207	1.305	1.419		
	1	1.143	1.107	1.206	1.293		
	1	1.203	1.122	1.318	1.516		
	1	1.140	1.199	1.139	1.248		
	1	1.105	1.157	1.183	1.372		
270s	1	1.200	1.267	1.414	1.470		
	1	1.087	1.085	1.202	1.248		
	1	1.248	1.211	1.386	1.522		
	1	1.047	1.205	1.127	1.230		
	1	1.161	1.231	1.314	1.363		
288s	1	1.030	1.181	1.327	1.393		
	1	1.225	1.174	1.260	1.465		
	1	1.108	1.132	1.309	1.461		
	1	1.110	1.169	1.149	1.165		
	1	1.153	1.204	1.233	1.325		
306s	1	1.140	1.243	1.456	1.450		
	1	1.153	1.157	1.281	1.379		
		1.175	1.090	1.346	1.434		
	1	1.161	1.249	1.186	1.274		
	1	1.138	1.272	1.261	1.419		
324s	1	1.190	1.243	1.378	1.577		
		1.193	1.157	1.290	1.360		
		1.250	1.090	1.428	1.524		
	1	1.104	1.249	1.161	1.195		
	1	1.150	1.272	1.270	1.333		
342s	1	1.110	1.297	1.412	1.532		
	1	1.200	1.131	1.236	1.452		
	1	1.259	1.171	1.402	1.642		
	1	1.135	1.232	1.130	1.201		
	1	1.133	1.185	1.178	1.338		
360s	1	1.140	1.219	1.328	1.457		
	1	1.106	1.076	1.160	1.391		
	1	1.231	1.175	1.383	1.607		
	1	1.156	1.263	1.184	1.229		
	1	1.171	1.225	1.224	1.344		
378s	1	1.210	1.299	1.394	1.611		
	1	1.132	1.157	1.294	1.312		
	1	1.234	1.091	1.308	1.521		
	1	1.181	1.263	1.219	1.285		
	1	1.087	1.164	1.218	1.332		
g							
0mM	0.25mM	0.5mM	1mM	2mM			
1.13	0.98	0.46	0.24	0.35			
1.40	1.35	0.98	0.52	0.44			
1.45	1.46	1.03	0.52	0.45			
i							
0	24h	48h	72h				
0.98	0.73	0.57	0.48				
1.12	1.18	0.58	0.53				
1.33	0.70	0.66	0.61				
k				l			
Ctrl	TMAO	TMAO + BAPTA	BAPTA	Ctrl	TMAO	TMAO+ BAPTA	BAPTA
0.87	1.62	0.82	0.87	0.51	1.45	0.67	0.50
0.88	1.69	1.04	1.06	1.00	1.89	1.05	0.86
0.48	1.72	1.38	0.99	0.70	1.56	0.88	0.93
n							
Ctrl	TMAO	TMAO+ BAPTA	BAPTA				
0.89	3.69	1.28	0.72				

0.67	3.00	1.56	0.92				
1.22	3.15	1.35	1.08				
1.22	2.94	1.29	0.84				
**Figure 3**							
**a**		c		**e**			
NC	SERCA2a^{OE}	NC	SERCA2a^{OE}	NC	SERCA2a^{OE}	NC+ TMAO	SERCA2a^{OE}+ TMAO
1.00	3.70	0.50	1.10	1.00	0.99	1.23	0.93
1.00	4.83	0.56	1.24	1.00	1.10	1.26	0.94
1.00	3.61	0.34	1.40	1.00	0.95	0.98	1.03
1.00	3.02			1.00	1.04	1.01	1.00
				1.00	0.86	1.08	1.06
				1.00	0.96	1.17	0.90
				1.00	0.93	1.18	0.92
				1.00	0.93	1.05	0.92
				1.00	0.92	1.05	0.96
**g**				**h**			
NC	SERCA2a^{OE}	NC+ TMAO	SERCA2a^{OE}+ TMAO	NC	SERCA2a^{OE}	NC+ TMAO	SERCA2a^{OE}+ TMAO
0.50	0.50	1.08	0.49	0.26	0.30	1.18	0.59
0.16	0.54	0.88	0.68	0.16	0.64	1.58	0.48
0.24	0.30	1.11	0.30	0.34	0.40	1.21	0.30
**i**							
NC	NC+TMAO	SERCA2a^{OE}	SERCA2a^{OE}+ TMAO				
0.89	2.23	0.74	0.99				
0.67	2.57	0.56	0.74				
1.22	3.04	1.01	1.35				
1.22	3.05	1.02	1.36				
**Figure 4**							
**b**							
Vehicle	TMAO	TMAO+ MG132 (250nm)	TMAO+ MG132 (500nm)	TMAO+ MG132(1 000nm)			
1.09	0.28	0.17	0.30	0.16			
1.38	0.59	0.32	0.31	0.39			
0.97	0.49	0.56	0.55	0.30			
**d**							
Vehicle	TMAO	TMAO+Baf （5uM）	TMAO+Baf （10uM）	TMAO +Baf （20uM			
1.11	0.28	0.83	0.92	2.18			
0.84	0.30	0.75	1.21	1.50			
0.90	0.11	0.65	1.16	2.35			
**f**							
Vehicle	TMAO	TMAO+CQ	CQ				
1.22	0.58	0.88	1.19				
1.46	0.75	1.50	1.38				
1.25	0.42	1.31	0.93				
**Figure 5**							
**c**							
0mM	0.25mM	0.5mM	1mM	2mM			
0.27	0.27	1.13	1.53	1.61			
0.32	0.33	1.60	1.73	1.86			
0.58	0.69	0.85	1.07	1.05			

**d**							
0mM	0.25mM	0.5mM	1mM	2mM			
0.37	0.57	1.03	1.53	1.61			
0.43	0.43	0.90	1.23	1.76			
0.59	0.69	0.85	1.07	1.45			
**e**							
0mM	0.25mM	0.5mM	1mM	2mM			
1.15	1.17	0.95	0.92	0.81			
1.16	0.98	0.77	0.81	0.83			
1.61	1.29	0.63	0.46	0.49			
**f**							
0mM	0.25mM	0.5mM	1mM	2mM			
0.37	0.67	1.03	1.53	1.61			
0.33	0.43	1.20	1.43	1.96			
0.59	0.69	1.05	1.27	1.65			
h				**i**			
Ctrl	TMAO	TMAO+3MA	3MA	Ctrl	TMAO	TMAO+ 3MA	3MA
1.02	0.14	1.03	0.98	0.22	0.63	0.26	0.36
1.22	0.44	0.83	0.68	0.32	0.73	0.36	0.46
0.92	0.34	1.03	0.88	0.42	0.83	0.46	0.56
**j**				**m**			
Ctrl	TMAO	TMAO+3MA	3MA	Ctrl	TMAO	TMAO+ BafA1	BafA1
2.00	15.00	6.00	3.00	3.00	15.00	55.00	13.00
3.00	16.00	7.00	11.00	4.00	17.00	52.00	15.00
8.00	26.00	5.00	1.00	6.00	19.00	57.00	17.00
				3.00	32.00	1.00	1.00
				4.00	35.00	2.00	2.00
				6.00	38.00	4.00	4.00
**n**				**p**			
Ctrl	TMAO	TMAO+BafA1	BafA1	Ctrl	TMAO	TMAO+ 3MA	3MA
3.00	13.00	42.00	6.00	0.93	1.90	0.86	0.58
5.00	17.00	45.00	7.00	0.99	2.33	0.91	0.59
2.00	19.00	50.00	8.00	1.04	1.89	1.22	1.02
4.00	40.00	50.00	7.00				
6.00	37.00	47.00	9.00				
3.00	35.00	50.00	10.00				
**r**				**t**			
Vehicle	TMAO	TMAO+3MA	3MA	Ctrl	TMAO	TMAO+ 3MA	3MA
1.30	0.51	1.34	1.01	0.13	1.07	0.45	0.48
1.58	0.80	1.49	1.40	0.33	0.72	0.51	0.30
1.49	0.28	1.27	1.27	0.47	0.98	0.38	0.57
**u**							
Ctrl	TMAO	TMAO+3MA	3MA				
0.43	0.84	0.43	0.38				
0.22	0.94	0.52	0.19				
0.33	1.07	0.34	0.27				
**Figure 6**							
**d**			**e**				
Ctrl	siCtrl	siATG5	Ctrl	siCtrl	siATG5		
1.15	1.10	0.45	1.10	1.20	0.40		
0.95	1.20	0.30	1.20	1.20	0.20		

0.90	1.00	0.40	1.30	1.00	0.30		
**h**				**i**			
siRNA	siATG5	siRNA+TMAO	siATG5+TMAO	siRNA	siATG5	siRNA+ TMAO	siATG5+ TMAO
0.80	0.88	0.26	0.69	0.80	0.26	1.08	0.26
0.64	0.98	0.14	0.78	0.64	0.24	0.98	0.34
0.60	0.71	0.21	0.50	0.60	0.21	0.81	0.27
**k**				**l**			
siRNA	siATG5	siRNA+TMAO	siATG5+TMAO	siRNA	siATG5	siRNA+ TMAO	siATG5+ TMAO
0.60	0.40	0.98	0.59	0.59	0.50	1.18	0.80
0.44	0.46	0.88	0.48	0.68	0.56	1.28	0.64
0.40	0.54	1.01	0.50	0.40	0.44	1.21	0.50
**m**							
siRNA	siATG5	siRNA+TMAO	siATG5+TMAO				
0.99	0.84	2.33	0.99				
0.77	0.66	2.67	0.84				
1.32	1.11	3.14	1.55				
1.32	1.12	3.15	1.56				
**Figure 7**							
**b**			**c**				
Ctrl	TMAO	TMAO+3MA	Ctrl	TMAO	TMAO+3MA		
71.00	54.00	74.00	34.00	23.00	37.00		
78.00	50.81	69.00	41.00	27.00	34.00		
71.00	52.00	70.00	34.00	30.00	34.00		
65.00	54.00	70.00	31.00	23.00	39.00		
**d**			**e**				
Ctrl	TMAO	TMAO+3MA	Ctrl	TMAO	TMAO+3MA		
4.35	5.30	4.99	6.32	8.19	6.25		
4.35	5.71	4.68	5.99	8.20	6.14		
4.30	5.20	4.90	5.70	7.66	6.23		
4.40	5.59	4.88	6.50	8.42	6.03		
4.46	5.51	4.50	7.11	8.39	5.16		
4.19	5.69	4.60	7.06	8.40	7.18		
4.45	5.62	4.92	6.91	7.80	6.42		
4.46	5.50	5.02	7.04	8.00	6.45		
**j**			**k**				
Ctrl	TMAO	TMAO+3MA	Ctrl	TMAO	TMAO+3MA		
0.30	0.82	0.39	1.42	0.35	1.63		
0.48	1.16	0.21	1.62	0.46	1.73		
0.40	0.92	0.41	1.30	0.58	1.30		
0.45	0.85	0.40	1.40	0.62	1.40		
0.25	1.12	0.25	1.53	0.54	1.53		
**l**			**n**				
Ctrl	TMAO	TMAO+3MA	Ctrl	TMAO	TMAO+3MA		
0.94	1.25	0.60	1.15	0.35	1.32		
0.65	1.48	0.70	1.34	0.28	0.95		
0.74	1.68	0.75	0.98	0.47	0.82		
0.81	1.32	0.55	1.00	0.31	1.31		
0.52	1.54	0.65	1.02	0.11	0.90		
			0.88	0.50	0.89		
**o**			**p**				
Ctrl	TMAO	TMAO+3MA	Ctrl	TMAO	TMAO+3MA		
0.48	1.21	0.18	0.44	0.83	0.48		
0.43	0.90	0.20	0.31	0.94	0.38		

0.45	1.20	0.24	0.22	1.35	0.21		
0.23	0.90	0.35	0.35	1.53	0.45		
0.30	1.10	0.41	0.45	0.84	0.35		
0.40	1.30	0.13					
**Figure S1**							
c			**d**				
Ctrl	ISO	ISO+TMAO	Ctrl	ISO	ISO+TMAO		
71.00	62.00	45.00	34.00	27.00	22.00		
78.00	61.00	42.00	41.00	28.00	18.00		
71.00	61.00	40.00	35.00	27.00	17.00		
65.00	65.00	46.00	30.00	26.00	16.00		
**e**			**f**				
Ctrl	ISO	ISO+TMAO	Ctrl	ISO	ISO+TMAO		
6.32	8.20	9.65	4.35	5.86	6.12		
5.99	8.19	9.64	4.35	5.62	6.27		
5.70	7.44	9.70	4.30	5.98	6.62		
7.26	7.57	8.24	4.71	5.93	6.50		
7.11	8.39	9.74	4.59	5.99	6.73		
7.06	8.17	8.94	4.69	5.97	7.09		
6.91	8.54	9.99	4.45	5.96	7.00		
7.04	7.52	8.69	4.62	5.90	6.48		
**g**			**j**				
Ctrl	ISO	ISO+TMAO	Ctrl	ISO	ISO+TMAO		
20.96	36.05	109.13	0.05	0.12	0.31		
12.31	50.79	69.84	0.06	0.15	0.23		
8.05	27.75	90.39	0.05	0.17	0.38		
10.38	41.95	86.25	0.05	0.20	0.20		
12.60	47.07	52.02	0.06	0.20	0.45		
15.13	62.40	55.12	0.07	0.12	0.30		
12.44	42.30	83.78					
12.78	41.27	73.94					
	44.74	56.30					
		144.53					
**k**							
Ctrl	ISO	ISO+TMAO					
0.31	0.54	1.06					
0.13	0.42	0.75					
0.29	0.58	0.99					
0.10	0.34	0.90					
0.25	0.48	0.95					
0.19	0.38	1.10					
**Figure S2**							
**c**				d			
Ctrl	Choline	Choline+DMB		Ctrl	Choline	Choline+DMB	
66.32	59.81	60.00		31.25	27.00	27.00	
64.00	59.00	72.00		30.00	26.80	30.00	
76.00	60.00	71.00		38.00	27.00	34.00	
65.00	57.00	65.00		35.00	25.00	32.00	
**e**				**f**			
Ctrl	Choline	Choline+DMB		Ctrl	Choline	Choline+DMB	
4.18	5.67	4.39		6.22	6.71	6.20	
4.70	5.57	4.42		6.35	6.90	6.10	
4.52	5.54	3.94		6.48	7.12	6.36	
4.09	5.66	4.41		5.82	7.08	6.41	
4.55	5.50	4.91		6.20	7.15	5.51	
4.72	5.24	4.82		6.01	6.91	5.91	
4.91	5.71	4.69		6.12	6.89	6.01	
4.56	5.01	4.51		6.30	7.20	6.19	

**g**				j			
Ctrl	Choline	Choline+DMB		Ctrl	Choline	Choline+DMB	
10.38	213.78	39.65		0.21	0.94	0.66	
12.60	332.01	29.73		0.33	0.82	0.43	
15.13	270.39	35.22		0.29	1.08	0.49	
12.44	199.46	46.30		0.11	0.82	0.31	
12.78	230.39	59.74		0.32	0.89	0.42	
12.44	253.78	27.57		0.21	1.01	0.11	
11.00	250.00	50.59					
13.00	260.00	45.00					

**k**		
Ctrl	Choline	Choline+DMB
0.31	1.34	0.46
0.23	0.72	0.43
0.13	0.98	0.59
0.21	1.80	0.41
0.15	1.51	0.32
0.12	1.39	0.49

**Figure S3**								
0mM	0.0625mM	0.125mM	0.25mM	0.5mM	1mM	2mM	4mM	8mM
1.00	0.99	1.01	1.00	0.96	0.97	0.96	0.97	0.95
1.00	1.01	1.01	1.01	1.02	0.99	0.91	0.86	0.79
1.00	1.06	1.01	1.05	1.03	1.05	1.08	1.11	1.11

**Figure S4**	
Ctrl	TMAO
1.00	0.70
1.00	0.83
1.00	0.61
1.00	0.62
1.00	1.01
1.00	0.91
1.00	0.90
1.00	1.01
1.00	0.92
