# Supplementary material for: The gut microbiota metabolite trimethylamine N-oxide promotes cardiac hypertrophy by activating the autophagic degradation of SERCA2a
Source: Commun Biol. 2025 Apr 10;8:596. doi: 10.1038/s42003-025-08016-9 (PMC11986001; doi:10.1038/s42003-025-08016-9)
Supplement: Supplementary file 5 — Reporting Summary [file 42003_2025_8016_MOESM5_ESM.pdf]

nature portfolio

Corresponding author(s): DBPR and COMMSBIO-22-3030C

Last updated by author(s): October 25[illegible]2024

Reporting Summary

Nature Portfolio wishes to improve the reproducibility of the work that we publish. This form provides structure for consistency and transparency in reporting. For further information on Nature Portfolio policies, see our Editorial Policies and the Editorial Policy Checklist.

Statistics

For all statistical analyses, confirm that the following items are present in the figure legend, table legend, main text, or Methods section.

n/a	Confirmed	
☐	☒	The exact sample size (*n*) for each experimental group/condition, given as a discrete number and unit of measurement
☐	☒	A statement on whether measurements were taken from distinct samples or whether the same sample was measured repeatedly
☐	☒	The statistical test(s) used AND whether they are one- or two-sided<br>*Only common tests should be described solely by name; describe more complex techniques in the Methods section.*
☐	☒	A description of all covariates tested
☐	☒	A description of any assumptions or corrections, such as tests of normality and adjustment for multiple comparisons
☐	☒	A full description of the statistical parameters including central tendency (e.g. means) or other basic estimates (e.g. regression coefficient) AND variation (e.g. standard deviation) or associated estimates of uncertainty (e.g. confidence intervals)
☐	☒	For null hypothesis testing, the test statistic (e.g. F, t, r) with confidence intervals, effect sizes, degrees of freedom and P value noted<br>*Give P values as exact values whenever suitable.*
☐	☒	For Bayesian analysis, information on the choice of priors and Markov chain Monte Carlo settings
☐	☒	For hierarchical and complex designs, identification of the appropriate level for tests and full reporting of outcomes
☐	☒	Estimates of effect sizes (e.g. Cohen's *d*, Pearson's *r*), indicating how they were calculated

Our web collection on statistics for biologists contains articles on many of the points above.

Software and code

Policy information about availability of computer code

Data collection	Bio-Rad CFX Manager 3.1, Bio Tek, stereomicroscope(Olympus SZ61),HT7700 transmission electron microscope, Vevo 2100 High-Resolution, Imaging System, Ultra performance Liquid Chromatography-Tandem Mass Spectrometry, Confocal microscope, Nikon Microsystem
Data analysis	Figi, Image Lab, GraphPad Prism8, SPSS 16.0

For manuscripts utilizing custom algorithms or software that are central to the research but not yet described in published literature, software must be made available to editors and reviewers. We strongly encourage code deposition in a community repository (e.g. GitHub). See the Nature Portfolio guidelines for submitting code & software for further information.

Data

Policy information about availability of data

All manuscripts must include a data availability statement. This statement should provide the following information, where applicable:

- Accession codes, unique identifiers, or web links for publicly available datasets
- A description of any restrictions on data availability
- For clinical datasets or third party data, please ensure that the statement adheres to our policy

The numerical source data used to generate the main figures have been uploaded as Supplementary Data 1. The western blot images utilized for figure generation can be found in the Supplementary Information Figure. S10. For any other data, please contact the corresponding author and they will be provided upon reasonable request.

Research involving human participants, their data, or biological material

Policy information about studies with human participants or human data. See also policy information about sex, gender (identity/presentation), and sexual orientation and race, ethnicity and racism.

Reporting on sex and gender	Not involved
Reporting on race, ethnicity, or other socially relevant groupings	Not involved
Population characteristics	Not involved
Recruitment	Not involved
Ethics oversight	Not involved

Note that full information on the approval of the study protocol must also be provided in the manuscript.

Field-specific reporting

Please select the one below that is the best fit for your research. If you are not sure, read the appropriate sections before making your selection.

☒ Life sciences ☐ Behavioural & social sciences ☐ Ecological, evolutionary & environmental sciences

For a reference copy of the document with all sections, see nature.com/documents/nr-reporting-summary-flat.pdf

Life sciences study design

All studies must disclose on these points even when the disclosure is negative.

Sample size	n=3-11 in each group. The results were presented as means ±SD(standard deviation). Statistical analysis was performed by unpaired Student's t-test for two comparisons or ANOVA followed by a post hoc Tukey's test for multiple comparisons.
Data exclusions	no data were excluded.
Replication	we confirm that all attempts at replication were successful.
Randomization	the all samples were random allocated into experimental groups.
Blinding	the investigators were blinded to group allocation during data collection and analysis.

Reporting for specific materials, systems and methods

We require information from authors about some types of materials, experimental systems and methods used in many studies. Here, indicate whether each material, system or method listed is relevant to your study. If you are not sure if a list item applies to your research, read the appropriate section before selecting a response.

Materials & experimental systems

n/a	Involved in the study	
☐	☒	Antibodies
☐	☒	Eukaryotic cell lines
☒	☐	Palaeontology and archaeology
☐	☒	Animals and other organisms
☒	☐	Clinical data
☒	☐	Dual use research of concern
☒	☐	Plants

Methods

n/a	Involved in the study	
☒	☐	ChIP-seq
☒	☐	Flow cytometry
☒	☐	MRI-based neuroimaging

Antibodies

Antibodies used	ANP (ab225844, lot number:GR336797 and GR3356779-2); Myh7 (ab172967, lot number:GR303508-14 and GR3337892-2) ; SERCA2a (ab3625, lot number:GR3247974-7) ; p62CST,NO.39749;LC3A/B (sc-271625,lot number:H0122 AND F2221); ATG5

	(sc-133158,lot number:K1521AND j0822), and LAMP1(sc-20011,lot number:C1022 AND H0922); ATG7 (ZENBIO 382798,lot number KK0928) and anti-Flag (ZENBIO,2501121,lot number LL0117) GAPDH (YEASEN:30202ES60,lot number G1908771)
Validation	ANP (ab225844):Species: Rat, Mouse; Application: WB 1:1000 . Myh7 (ab172967):Species: Rat, Mouse; Application: WB 1:1000 . SERCA2a (ab3625):Species: Rat, Mouse; Application: WB 1:5000 . p62(CST,NO.39749,):Species: Rat, Mouse; Application: WB 1:1000 . LC3A/B (sc-271625,):Species: Rat, Mouse; Application: immunofluorescence staining 1:20 . ATG5 (sc-133158,,):Species: Rat, Mouse; Application: immunofluorescence staining 1:20),WB 1:100),CO-IP(1:20). LAMP1(sc-20011):Species: Rat, Mouse; Application: immunofluorescence staining 1:20 . anti-Flag(ZENBIO,2501121,):Species: Rat, Mouse; Application: WB 1:100),CO-IP(1:20). GAPDH(YEASEN,30202ES60):Species: Rat, Mouse; Application: WB 1:5000

Eukaryotic cell lines

Policy information about cell lines and Sex and Gender in Research

Cell line source(s)	H9c2 cells lines were purchased from Procell and cultures in a DMEM medium. Neonatal rat primary cardiomyocytes were isolated from the ventricles of neonatal male Sprague-Dawley rats(2-3 days old) through enzyme digestion.
Authentication	each cell line has STR authentication ceritificate.
Mycoplasma contamination	we conform that all cell lines tested negative for mycoplasma contamination.
Commonly misidentified lines (See ICLAC register)	no cell lines used in this study were found in the database of commonly misidentified cell lines

Animals and other research organisms

Policy information about studies involving animals; ARRIVE guidelines recommended for reporting animal research, and Sex and Gender in Research

Laboratory animals	Male C57BL/6 mice (6 weeks old) weighing between 18 and 20g were obtained from Hunan SJA Laboratory Animal Co. Ltd (China). Animals were kept in a constant temperature condition with a 12-h light/dark cycle and provided with standard laboratory chow and tap water.
Wild animals	the study did not involved wild animals.
Reporting on sex	In order to reduce the randomness of the experiment and the impact of estrogen on the stability of the model, we chose male mice for the experiment.
Field-collected samples	The study did not involve samples collected from the field.
Ethics oversight	All procedures and experimental protocols were conducted according to the National Institutes of Health Guide for the Care and Use of Laboratory Animals and approved by the Animal Care and Use Committee of the Hunan Normal University. Research proposal number:D2021041.

Note that full information on the approval of the study protocol must also be provided in the manuscript.

Plants

Seed stocks	Not involved
Novel plant genotypes	Not involved
Authentication	Not involved
